# Cost Utility Analysis of Multidisciplinary Postacute Care for Stroke: A Prospective Six-Hospital Cohort Study

**DOI:** 10.3389/fcvm.2022.826898

**Published:** 2022-03-30

**Authors:** Yu-Ching Chen, Yu-Jo Yeh, Chung-Yuan Wang, Hsiu-Fen Lin, Ching-Huang Lin, Hong-Hsi Hsien, Kuo-Wei Hung, Jung-Der Wang, Hon-Yi Shi

**Affiliations:** ^1^Department of Healthcare Administration and Medical Informatics, Kaohsiung Medical University, Kaohsiung, Taiwan; ^2^Department of Public Health, College of Medicine, National Cheng-Kung University, Tainan, Taiwan; ^3^Department of Physical Medicine and Rehabilitation, Pingtung Christian Hospital, Pingtung, Taiwan; ^4^Department of Nursing, Meiho University, Pingtung, Taiwan; ^5^Department of Neurology, Kaohsiung Medical University Hospital, Kaohsiung, Taiwan; ^6^Department of Neurology, Kaohsiung Medical University, Kaohsiung, Taiwan; ^7^Division of Neurology, Kaohsiung Veterans General Hospital, Kaohsiung, Taiwan; ^8^Department of Internal Medicine, St. Joseph Hospital, Kaohsiung, Taiwan; ^9^Division of Neurology, Department of Internal Medicine, Yuan's General Hospital, Kaohsiung, Taiwan; ^10^Department of Medical Research, Kaohsiung Medical University Hospital, Kaohsiung, Taiwan; ^11^Department of Medical Research, China Medical University Hospital, China Medical University, Taichung, Taiwan; ^12^Department of Business Management, National Sun Yat-sen University, Kaohsiung, Taiwan

**Keywords:** postacute care, cost-utility, stroke, incremental cost-utility ratios, cost saving

## Abstract

**Background:**

Few studies have compared the optimal duration and intensity of organized multidisciplinary neurological/rehabilitative care delivered in a regional/district hospital with the standard rehabilitative care delivered in the general neurology/rehabilitation ward of a medical center. This study measured functional outcomes and conducted cost-utility analysis of an organized multidisciplinary postacute care (PAC) project in secondary care compared with standard rehabilitative care delivered in tertiary care.

**Methods:**

This prospective cohort study enrolled 1,476 patients who had a stroke between March 2014 and March 2018 and had a modified Rankin scale score of 2–4. After exact matching for age ± 1 year, sex, year of stroke diagnosis, nasogastric tube, and Foley catheter and propensity score matching for the other covariates, we obtained 120 patients receiving PAC (the PAC group) from four regional/district hospitals and 120 patients not receiving PAC (the non-PAC group) from two medical centers.

**Results:**

At baseline, the non-PAC group showed significantly better functional outcomes than the PAC group, including EuroQol-5 dimensions (EQ-5D), Mini-Mental State Examination (MMSE) and Barthel index (BI). During weeks 7–12 of rehabilitation, improvements in all functional outcomes were significantly larger in the PAC group (*P* < 0.001) except for Functional Oral Intake Scale (FOIS). Cost-utility analysis revealed that the PAC group had a significantly lower mean (± standard deviation) of direct medical costs (US$3,480 ± $1,758 vs. US$3,785 ± $3,840, *P* < 0.001) and a significantly higher average gain of quality-adjusted life years (0.1993 vs. 0.1233, *P* < 0.001). The PAC project was an economically “dominant” strategy.

**Conclusions:**

The PAC project saved costs and significantly improved the functional outcomes of patients with stroke with slight to moderately severe disabilities. Randomized control trials are required to corroborate these results.

## Introduction

Stroke is the second leading cause of death and also the second highest burden estimated with disability-adjusted life-years worldwide (1). In Taiwan, which has a population of approximately 23 million people, stroke is the third leading cause of death and most common cause of complex disability (2). The percentage of patients with disability at 1 and 6 months after the first incident of stroke was 61.2 and 51.72%, respectively (3). In addition, 10.4% of Taiwanese patients with acute stroke had a prolonged hospital stay, which accounted for 47.8% of the total in-hospital medical expenses for stroke (4). In the United States, 59.1% to 82.1% patients hospitalized for stroke required post-acute care within 30 days after discharge (5). Stroke patients treated in an inpatient rehabilitation facility experienced shorter length of rehabilitation stay, less emergency room utilization and lower mortality, but incurred higher cost, than those receiving rehabilitation in a skilled nursing facility (6).

Based on measures of EQ-5D (7), MMSE (8), BI (9), Instrumental Activities of Daily Living Scale (IADL) (10), rehabilitation to improve quality of life emphasizes training/re-training on functional and daily activities including self-care, mobility, cognitive skills, and psychosocial skills, which could actually be accomplished by a multidisciplinary PAC project. To our knowledge, cost-utility analysis of PAC has rarely been prospectively investigated (11). In addition, few studies seem to have compared the optimal duration and intensity of organized multidisciplinary neurological/rehabilitative care delivered in a regional/district hospital (secondary care) vs. standard rehabilitative care delivered in the general neurology/rehabilitation ward of a medical center (tertiary care). Launched in 2014 by the National Health Insurance (NHI) of Taiwan to contain PAC cost without compromising functional outcomes, the PAC project was executed to enroll stroke patients with slight to moderately severe disability and potential for active rehabilitation.

Therefore, the objective of this prospective cohort study is to measure functional outcomes and conduct cost-utility analysis of an organized multidisciplinary PAC project in secondary care compared with standard rehabilitative care delivered in tertiary care.

## Materials and Methods

### The Post-acute Care-Cerebrovascular Diseases Project

A PAC-CVD project was launched in Taiwan in 2014 to contain PAC cost by two approaches: (I) across-level transfer of patients in post-acute phase of stroke from neurology wards in medical centers (tertiary care) to neurology/rehabilitation wards in regional and district hospitals (secondary care); (II) within-level transfer of patients from neurology wards in regional/district hospitals (secondary care) to neurology/rehabilitation wards in regional/district hospitals (secondary care). The PAC-CVD project was designed to improve functional outcomes by organizing a multidisciplinary team, which included neurologists, physiatrists, physiotherapists, occupational therapists, speech therapists and registered nurses. The average number of days from stroke onset to PAC ward admission was significantly shorter in patients transferred within level (9.88 days) compared to those transferred across level (17.11 days) (12). The 12-week PAC-CVD project delivered in regional/district hospitals (secondary care) featured more reimbursement and higher intensity of rehabilitation compared to non-PAC care delivered in medical centers (tertiary care). The reimbursement schedule of the PAC project (per diem) in this study is summarized as follows: NT$3,486 (US$113) per day if 3–5 sessions a day within 12 weeks; physical therapy: 1–2 sessions per weekday, 30–60 min each session, and 1 session per weekend, 30–60 min each session; occupational therapy: 1–2 sessions per weekday, 30–60 min each session and 1 session per weekend, 30–60 min each session; speech therapy: at least 5 sessions a week, depending on how well a patient can communicate and/or swallow. The reimbursement of standard rehabilitation for non-PAC care (fee for service): NT$600 (US$19.4) per session; 1 session per weekday for physical therapy, occupational therapy and/or speech therapy, respectively, with 30–60 min each session. The longest duration of non-PAC care usually allows for hospitalization of 28–42 days after acute stroke by National Health Insurance. Details of the PAC-CVD project are described in the Supplementary Material.

### Study Design and Sample

**Figure 1:**
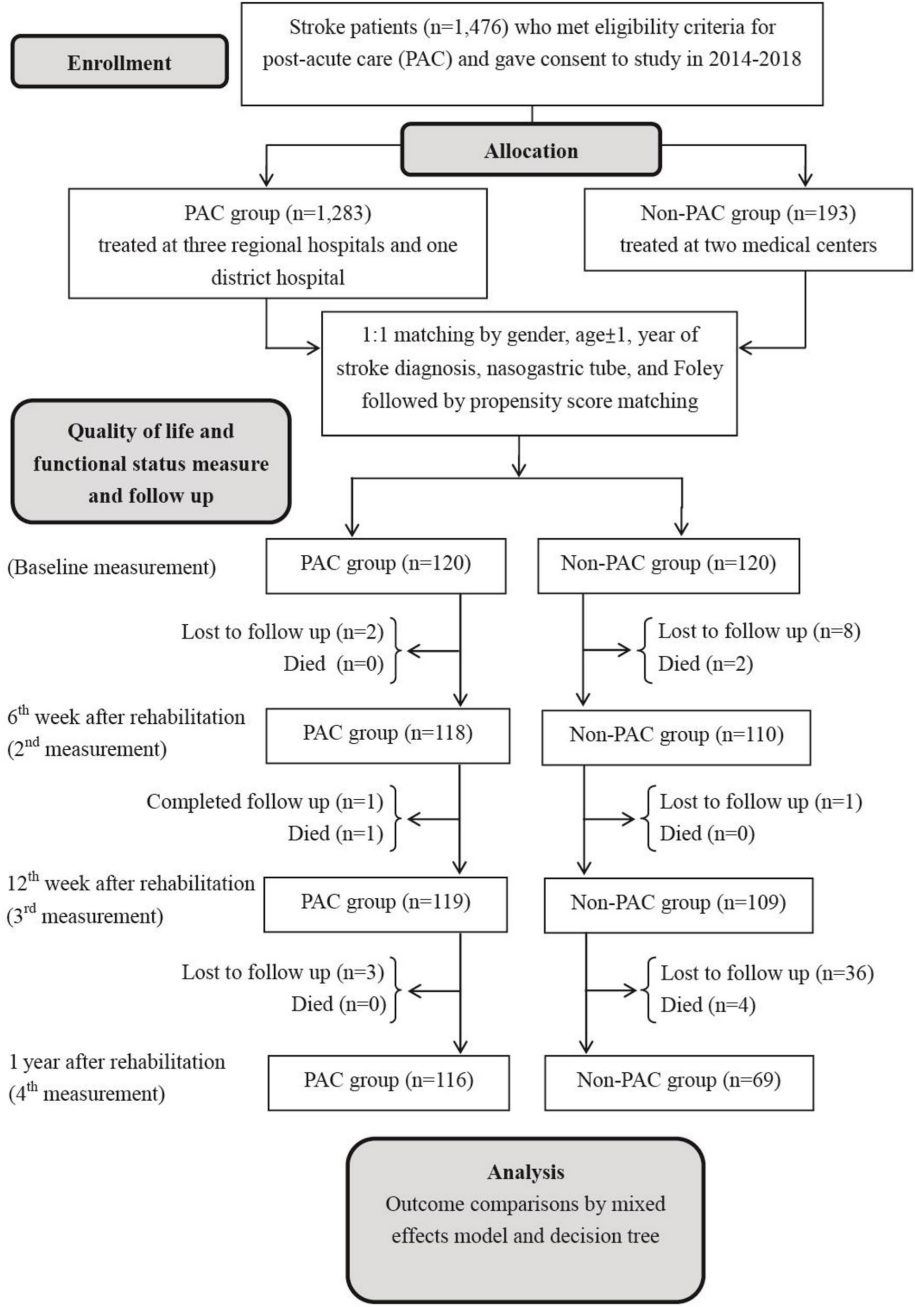
Flowchart of sample selection for prospective cohort analysis.

This is a prospective cohort study to evaluate the cost-utility of multidisciplinary post-acute care project (vs. standard rehabilitation care) for patients with stroke, which is defined as ICD-9-CM codes 433.xx, 434.xx, and 436 for ischemic stroke and codes 430 and 431 for hemorrhagic stroke; for their counterparts in ICD-10-CM, please see Supplementary Table 1. Patients were admitted to a PAC ward at one of four hospitals (three regional hospitals and a district hospital) or to a non-PAC ward at two medical centers in southern Taiwan between March 2014 and March 2018. The inclusion criteria were: (I) diagnosis of acute stroke; (II) stroke onset day within 30 days; and (III) modified Rankin Scale (MRS) scores of 2, 3, and 4, corresponding to slight, moderate, and moderately severe disability, respectively (13). The PAC project was a national health policy and stroke patients were allocated into either group at the discretion of the physician-in-charge after shared decision-making. Figure 1 is a flow diagram of the study procedure, which features enrollment, allocation, repeated measures of functional status outcomes, and analysis.

### Economic Evaluation

The costs of both treatment options over 1 year were compared, using the direct-cost approach.

### Outcome Measures

The EuroQol-5 dimensions (EQ-5D) questionnaire is a preference-based, generic and self-reported instrument that can help to understand the impact of stroke, and provides a utility value based upon mobility, self-care, usual activities, pain/ discomfort and anxiety/depression, each of which has three levels of severity (7, 14). The Mini-Mental State Examination (MMSE) is a widely used cognitive function test for the elderly, which includes tests of orientation, attention, memory, language and visual-spatial skills (8). The Barthel Index (BI) score is used to measure functional disability in daily self-care activities (e.g., bowels, bladder, grooming, toilet use, feeding, transfer, mobility, dressing, stairs and bathing) (9). The Lawton-Brody Instrumental Activities of Daily Living Scale (IADL) involves eight tasks: telephone use, shopping, meal preparation (C), housekeeping (D), laundry (E), use of transportation, responsibility for medication intake and handling finances (10). Tasks C, D and E are excluded when assessing men. The Functional Oral Intake Scale (FOIS) is used to assess functional oral intake in stroke patients with dysphagia (15). The FOIS classifies swallowing function from level 1 (nothing by mouth) to level 7 (total oral diet with no restrictions). The Berg Balance Scale (BBS) is a scale of functional balance, including static and dynamic balance (16). Each item on this 14-item scale is rated from 0 (poor balance) to 4 (good balance) with maximum score of 56. When overall utility, based on EQ-5D, serves as the dependent variable, all the variance inflation factors (VIFs) are <5, indicating negligible or acceptable multicollinearity (17). Therefore, we allow BI and BBS to show the robustness of our results. All enrolled patients were scheduled to complete repeated measures of the EuroQol-5 dimensions (EQ-5D) questionnaire and the other five functional outcomes at four time points: at baseline, at the end of the 6th week and 12th week of rehabilitation, and at the end of one year.

### Estimation of Cost

In accordance with the reimbursement criteria established by the National Health Insurance Administration (NHI), direct costs included fees for physician, laboratory, pharmacy, procedures, and rehabilitative therapy, etc. All cost inputs were adjusted to 2019 U.S. dollars and discounted annually by 3%.

### Estimation of Utility

To estimate quality-adjusted life-years (QALYs), cost-utility analysis often uses “utility scores” (health state valuations) anchored by 0 and 1, where 0 indicates death and 1 indicates full health. This study used the time trade-off valuation procedure to convert EQ-5D total scores to utility. The cost-utility of PAC for stroke patients was estimated using QALYs (14, 18).

The two components used to calculate QALYs are the gain in quality of life and the number of life years over which the gain has been sustained. In this study, because the number of year remained at 1, QALYs were calculated based on a measure of utility derived from EQ-5D. The utilities reported by each participant were multiplied by the assumed duration of sustained benefit after intervention (summed up to the end of 1 year) to estimate the number of QALYs. To maintain consistency with the QALYs calculation, this study assumed that the only resources used by patients were those captured during the 1 year of follow-up. That is, the analysis assumed that patient did not incur any other healthcare costs during the remainder of the year.

### Statistical Analysis

To minimize the potential selection bias, firstly, the patients were selected through exact matching for the following variables: age ± 1 year, gender, year of stroke diagnosis, nasogastric tube and Foley catheter. Next, the propensity score matching (PSM) approach was used to minimize baseline differences in education, body mass index, stroke type, hypertension, diabetes mellitus, hyperlipidemia, atrial fibrillation, and previous stroke. The choice of matching algorithm was greedy nearest neighbor matching; within a matched pair, we chose a caliper width of within 0.2 of the standard deviation of the logit score; matching ratio of PAC-to-non-PAC patients was 1 to 1; and the matching was run without replacement (19). Finally, 120 patients each in the PAC and non-PAC group were obtained (Table 1).

**Table 1:** Distributions of patient characteristics before and after matching by demographic characteristics and by propensity scores.

**Variables**		**Before matching**			**After matching**		
		**PAC (*n* = 1,283)**	**Non-PAC (*n* = 193)**	***P* value**	**PAC (*n* = 120)**	**Non-PAC (*n* = 120)**	***P* value**
Cerebrovascular accident, year of diagnosis	2014	221 (17.2%)	0 (0%)		0 (0%)	0 (0%)	
	2015	302 (23.5%)	15 (7.8%)		11 (9.17%)	11 (9.17%)	
	2016	392 (30.6%)	107 (55.7%)	<0.001	66 (55%)	66 (55%)	1
	2017	363 (28.3%)	70 (36.5%)		43 (35.83%)	43 (35.83%)	
	2018	5 (0.4%)	0 (0%)		0 (0%)	0 (0%)	
Age, years^†^		65.16 ± 12.84	68.28 ± 13.95	0.002	67.45 ± 12.15	67.5 ± 12.27	0.975
Stroke patients, No. (%)		1,283 (100%)	193 (100%)	<0.001	120 (100%)	120 (100%)	1
Gender (% male)		800 (62.4%)	122 (63.2%)	0.880	81 (67.5%)	81 (67.5%)	1
Nasogastric tube, No. (%)		233 (18.2%)	56 (29.0%)	0.001	11 (9.17%)	11 (9.17%)	1
Foley catheter, No. (%)		96 (7.5%)	38 (19.7%)	<0.001	8 (6.67%)	8 (6.67%)	1
Education, years^†^		8.95 ± 1.30	8.64 ± 4.85	0.370	8.84 ± 1.73	8.96 ± 4.85	0.804
BMI, kg/m^†^		24.03 ± 2.53	24.16 ± 3.46	0.630	24.22 ± 2.26	24.13 ± 3.4	0.815
Stroke type, Ischemic (%)		1,048 (81.7%)	176 (91.2%)	0.003	110 (91.67%)	109 (90.83%)	0.819
Hemorrhagic (%)		235 (18.3%)	17 (8.8%)		10 (8.33%)	11 (9.17%)	
Hypertension, No. (%)		890 (69.4%)	137 (71.0%)	0.710	78 (65%)	80 (66.67%)	0.785
Diabetes mellitus, No. (%)		499 (38.9%)	71 (36.8%)	0.630	48 (40%)	44 (36.67%)	0.595
Hyperlipidemia, No. (%)		463 (36.1%)	46 (23.8%)	0.001	37 (30.83%)	30 (25%)	0.314
Atrial fibrillation, No. (%)		106 (8.3%)	16 (8.3%)	0.990	8 (6.67%)	8 (6.67%)	1
Previous stroke, No. (%)		178 (13.9%)	48 (24.9%)	<0.001	17 (14.17%)	17 (14.17%)	1

*PAC, post-acute care; BMI, body mass index*.

† *Values are expressed as mean ± standard deviation*.

For repeated assessments within individual subjects, a linear mixed effects model was constructed for EQ-5D, MMSE, BI, IADL, FOIS, and BBS, respectively, with major determinants as fixed effects. The utility values estimated by EQ-5D and the scores for MMSE, BI, IADL, FOIS and BBS served as the dependent variables. Predictor/confounder variables used in this statistical model included PAC (PAC vs. non-PAC), measures of functional outcomes at four time points, year of stroke diagnosis, age, gender (male vs. female), nasogastric tube (yes vs. no), Foley catheter (yes vs. no), education, body mass index, stroke type (ischemic type vs. hemorrhagic type), and comorbidities (yes vs. no). A negative coefficient denoted that the variable predicted a worse functional status score, with the magnitude representing the effect. Effect size was obtained by using the Cohen d statistic, i.e., the difference in the mean post-intervention value minus the mean pre-intervention value divided by the pooled standard deviation (Supplementary Table 4) (20). Given the large number of patients lost to follow up in the non-PAC group, the robustness of the results was evaluated by another PSM of 116 PAC subjects to 69 non-PAC subjects at the end of 1 year. Sixty two patients each in the PAC and non-PAC group were obtained.

After converting EQ-5D scores into utility values, the number of QALYs over a period of 1 year was calculated for each participant using the area under the curve approach with control for imbalances in baseline utility scores (21). A *t*-test was performed to compare mean direct medical costs between the two groups. The incremental cost utility ratio (ICUR) was calculated as the ratio of the difference in mean costs per patient to the difference in mean QALYs per patient between PAC and non-PAC groups. A willingness-to-pay (WTP) threshold of gross domestic product (GDP) US$26,263.5 per QALY was used to assess cost-effectiveness. A project is termed an economically dominant strategy when it is both clinically superior and cost saving. To derive the cost-effectiveness acceptability curve, this study performed nonparametric bootstrapping on the incremental cost and effectiveness with 1,000 replications and Supplementary Figure 4 presents the results (22–24). The Statistical Analysis System^®^ software version 9.4 (SAS Institute, Cary, NC, USA) was used for statistical analyses. All *P* values reported were two-sided, and a *P* value of <0.05 was considered statistically significant.

### Sensitivity Analysis

For sensitivity testing, 164 patients in the PAC group and 82 in the non-PAC group were matched successfully. Comparisons of the two groups over 1 year revealed that the PAC group showed significantly greater improvement in EQ-5D and all functional outcomes, except for FOIS, where *p*-value for trend was 0.78 (Supplementary Table 7, Supplementary Figure 5). This study also conducted cost-utility analysis of PAC (*n* = 164) and non-PAC (*n* = 82) at the end of 1 year after stroke rehabilitation (Supplementary Table 8).

## Results

**Figure 2:**
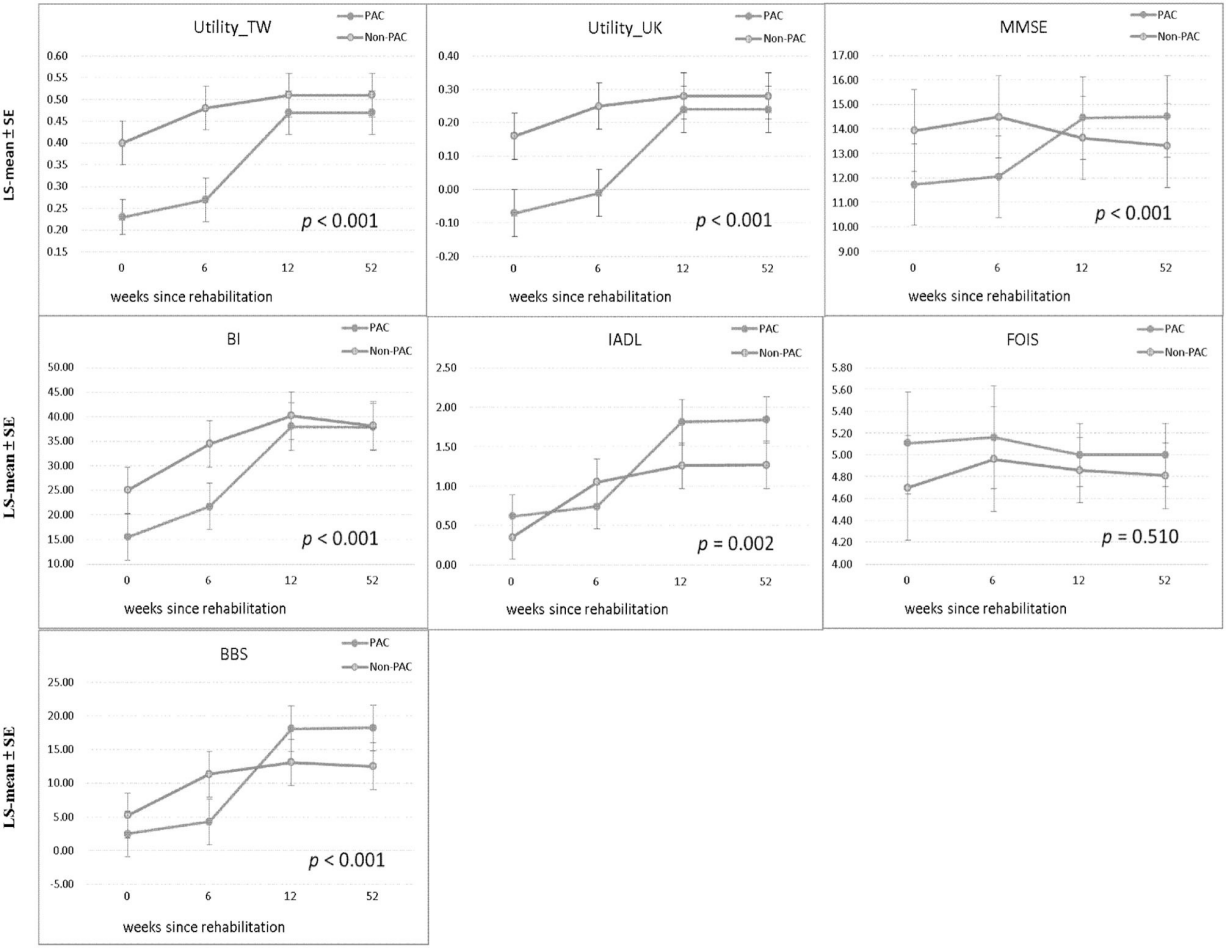
Comparison of LS-mean ± SE of each functional status measure between PAC and non-PAC groups at baseline, weeks 6 and 12, and 1 year and comparison of trend in each functional status measure between the groups. Utility_TW, utility (Taiwan); Utility_UK, utility (United Kingdom); MMSE, Mini-Mental State Examination; BI, Barthel Index; IADL, Instrumental Activities of Daily Living Scale; FOIS, Functional Oral Intake Scale; BBS, Berg Balance Scale; PAC, postacute care; LS-mean, least squares mean; SE, standard error. *P* values for trend in each functional status measure between the groups (120:120).

Table 1 compares the baseline characteristics of patients receiving PAC project with those of matched patients receiving standard rehabilitation. As presented in Table 2, at baseline, compared with the non-PAC group, the PAC group had significantly lower mean scores for EQ-5D utility (0.23 vs. 0.40; *P* < 0.0001), for cognitive function measured by MMSE (11.72 vs. 13.92; *P* = 0.020) and for self-care activities measured by BI (15.49 vs. 25.05; *P* = 0.001). The scores for IADL, FOIS, or BBS did not significantly differ between the two groups. When T_0_ values were used as reference values, the non-PAC group had larger improvements in BI, IADL and BBS than the PAC group at the end of the 6^th^ week (Supplementary Table 2). When T1 values were used as reference values, the PAC group achieved significantly larger improvements in the EQ-5D and in all functional outcomes (*P* < 0.001) except for the FOIS score during weeks 7–12 of rehabilitation than the non-PAC group (Supplementary Table 2). When T2 values were used as reference values, the two groups did not significantly differ in any functional outcome measures obtained at T3 when rehabilitation had ended at T2. During weeks 1–12, the PAC group had larger improvements in the EQ-5D and in MMSE, BI, and BBS scores than did the non-PAC group after controlling for baseline values (Supplementary Figure 1 and Supplementary Table 3). Overall, the PAC group exhibited a significantly better trend of improvement over the non-PAC group in the least squares mean scores of functional outcome measures, except for the FOIS score, over the 1-year duration of the study (*P* < 0.001; Table 2 and Figure 2). Supplementary Tables 5, 6 present the results for 62 patients each in the PAC and non-PAC group. Furthermore, compared with the non-PAC group (*n* = 62), the PAC group (*n* = 62) had significantly larger improvements in all outcomes, including the FOIS score (Supplementary Figure 2).

**Table 2:** Comparison of functional status trends between PAC and non-PAC groups after matching (120:120).

**Outcomes**		**Baseline (T0)**		**6^*th*^ week after rehabilitatio (T1)**		**12^*th*^ week after rehabilitation (T2)**		**1^*st*^ year after rehabilitatio (T3)**		***P* value for trend^¶^**
		**LS-mean ±S E**	***P* value^†^**	**LS-mean ±SE**	***P* value^†^**	**LS-mean ±SE**	***P* value^†^**	**LS-mean ±SE**	***P* value^†^**	
Utility_TW	PAC	0.23 ± 0.04	<0.001	0.27 ± 0.05	<0.001	0.47 ± 0.05	0.260	0.47 ± 0.05	0.260	<0.001
	Non-PAC	0.40 ± 0.05		0.48 ± 0.05		0.51 ± 0.05		0.51 ± 0.05		
Utility_UK	PAC	−0.07 ± 0.07	<0.001	−0.01 ± 0.07	<0.001	0.24 ± 0.07	0.300	0.24 ± 0.07	0.310	<0.001
	Non-PAC	0.16 ± 0.07		0.25 ± 0.07		0.28 ± 0.07		0.28 ± 0.07		
MMSE	PAC	11.72 ± 1.66	0.020	12.05 ± 1.67	0.010	14.44 ± 1.68	0.420	14.50 ± 1.67	0.240	<0.001
	Non-PAC	13.92 ± 1.67		14.48 ± 1.68		13.62 ± 1.70		13.31 ± 1.71		
BI	PAC	15.49 ± 4.69	0.001	21.76 ± 4.69	<0.001	38.02 ± 4.79	0.450	37.94 ± 4.81	0.930	<0.001
	Non-PAC	25.05 ± 4.72		34.49 ± 4.73		40.26 ± 4.84		38.22 ± 4.91		
IADL	PAC	0.62 ± 0.27	0.070	0.74 ± 0.28	0.130	1.81 ± 0.29	0.010	1.84 ± 0.29	0.010	0.001
	Non-PAC	0.35 ± 0.27		1.05 ± 0.29		1.26 ± 0.29		1.27 ± 0.30		
FOIS	PAC	5.11 ± 0.47	0.470	5.16 ± 0.47	0.720	5.00 ± 0.29	0.400	5.00 ± 0.29	0.280	0.510
	Non-PAC	4.70 ± 0.48		4.96 ± 0.48		4.86 ± 0.30		4.81 ± 0.30		
BBS	PAC	2.48 ± 3.34	0.140	4.29 ± 3.37	0.001	18.08 ± 3.40	0.020	18.23 ± 3.40	0.010	<0.001
	Non-PAC	5.22 ± 3.37		11.37 ± 3.4		13.09 ± 3.44		12.53 ± 3.46		

*PAC, post-acute care; Utility_TW, utility (Taiwan); Utility_UK, utility (United Kingdom); MMSE, Mini-Mental State Examination; BI, Barthel index; IADL, Instrumental Activities of Daily Living; FOIS, Functional Oral Intake Scale; BBS, Berg Balance Scale; LS-mean, least squares mean; SE, standard error;*

† *Each functional status measure was compared btween the PAC and non-PAC groups at baseline and after 6, 12, and 52 weeks*.

¶ *Trends in differences between PAC and non-PAC groups for each functional status measure during the study period*.

The mean direct medical cost per patient was US$3,480 in the PAC group and US$3,785 in the non-PAC group (Table 3). Cost-utility analysis revealed that the PAC treatment had a higher effectiveness (QALYs gain of 0.076) and a lower cost (cost reduction of US$305 ± US$2,986) than the non-PAC treatment. The ICUR was cost saving at US$ −4,013 per QALY, demonstrating that the PAC project was an economically dominant strategy. At a WTP threshold of US$26,263.5 per QALY, the PAC project had a 100% likelihood of being cost-effective compared to standard rehabilitation. For each set of 1,000 bootstrap resamples, Supplementary Figure 3 presents the corresponding cost-effectiveness plane, with incremental mean total direct medical cost on the y-axis, and incremental mean QALYs on the x-axis. All bootstrap observations were located in the southeast quadrant of the cost-effectiveness plane. Supplementary Figure 4 illustrates the cost-utility acceptability curve. The stochastic uncertainty associated with the mean incremental cost-effectiveness ratio indicated that our findings were robust.

**Table 3:** Cost-utility analysis of PAC and non-PAC groups within 1 year after stroke rehabilitation (120:120).

	**PAC group** **(*n* = 120)**	**Non-PAC group (*n* = 120)**	**Incremental difference**
	**mean ±SD (%)**	**mean ±SD (%)**	**(PAC-non-PAC)^¶^mean ±SD (%)**
**Baseline**			
Utility score	0.40 ± 0.18	0.57 ± 0.24	–(0.17 ± 0.21)^c^
**1 year after stroke rehabilitation**			
NHI total direct medical cost^†^	3,480 ± 1,758	3,785 ± 3,840	–(305 ± 2,986)
Utility score	0.63 ± 0.26	0.74 ± 0.26	–(0.10 ± 0.26)*^#^*
QALYs gained^§^	0.1993	0.1233	0.0760
ICUR (PAC – non-PAC)	dominant		−4,013

*PAC, post-acute care; mean, arithmetic mean; SD, standard deviation; NHI, national health insurance; QALYs, quality adjusted life years; ICUR, incremental cost-utility ratio*.

† *Mean direct cost for the PAC group. Per diem reimbursement packages received by hospitals varied by intensity of rehabilitation, e.g., per diem reimbursement for high-intensity rehabilitative care was the maximal packaged reimbursement of NT$3,587; per diem reimbursement for usual rehabilitative care was NT$2,411 (2019 exchange rate, NT $30.5 = US $1). Reimbursement included fees for physician, ward service, nursing, laboratory, rehabilitation therapy, and medication/pharmacy service fee, etc*.

§ *Area under the curve with control for baseline utility*.

¶ *P <0.001 for independent t test of the two groups*.

# *P = 0.01 for independent t test of the two groups*.

## Discussion

Although PAC has been promoted for the past 3 decades (25), evidence of its cost-effectiveness has been limited (11). To address this issue, we used exact and propensity score matching in this study. Although we observed that the PAC project saved cost compared with standard rehabilitation among stroke patients with an MRS scores 2–4, it did not necessarily imply that such an association was causal. However, we have the following arguments to corroborate this hypothesis: First, since we have controlled potential confounding factors including age, gender, year of stroke diagnosis, nasogastric tube, Foley catheter, and other covariates, such as hypertension (26), diabetes and atrial fibrillation etc. in the mixed effect model, the above factors cannot be explicable for the difference between PAC group and non-PAC group. Second, at the end of the one-year follow-up, only 69 subjects stayed in non-PAC group, while 116 subjects in PAC group remained, indicating a lower rate of adherence to standard rehabilitation. In further analysis, 69 non-PAC patients were matched by propensity score with 116 PAC patients, which resulted in 62 patients in each group (Supplementary Table 5). Compared with patients receiving standard rehabilitation, the patients receiving PAC consistently achieved significant improvement in all functional outcomes, including the FOIS score (*P* = 0.020, Supplementary Table 6). This further increased the robustness of positive outcomes shown in patients receiving PAC. Third, at baseline, the non-PAC group had statistically significant higher scores in EQ-5D utility, the MMSE, and the BI (*P* < 0.05); at 1 year, the outcome measures including EQ-5D utility, MMSE, BI, IADL, and BBS, showed a consistent better improvement in patients under PAC project than those receiving standard rehabilitation (*P* < 0.001) (Table 2). Finally, direct medical costs were lower for patients receiving PAC project than for those receiving standard rehabilitation (Table 3). Therefore, we tentatively concluded that PAC project saved cost compared with standard rehabilitation for mild to moderate stroke patients, and the difference could not be attributed to any known alternative cause.

Another major issue to be addressed is whether the sampled 120 patients receiving standard rehabilitation accurately represented all 193 non-PAC patients. Although this cohort was enrolled from stroke patients with modified Rankin scale 2–4, the PAC project has been a national policy, and only 13% (193/1476) of them were assigned to standard rehabilitation, particularly those with moderate severity. Among stroke patients, having a nasogastric tube, a retained Foley catheter, or a previous stroke was associated with impaired cognition and resilience, which was demanding in rehabilitation, and these conditions were present in approximately 29, 20, and 25%, respectively, of the non-PAC group, making these patients less likely to be matched with those in the PAC group (Table 1). To improve the comparability for rehabilitation at initial stage, we only found 120 pairs. Thus, the selectivity of our final matched samples may limit the generalizability of our findings to stroke patients with better rehabilitation potential, but still demonstrates the causal validity of cost-effectiveness of PAC project.

Lacking measure of functional outcomes in the multifaceted quality improvement intervention of stroke care, Pan et al. reported that the intervention gained 0.013 QALYs at an additional cost of US $140 in the first year, yielding an ICER of US $11,120 per QALY gained (11). The intervention was cost-effective in the first year, and more so in the second year at US$ 9,200 per QALY gained; our PAC project gained 0.076 QALYs at a negative cost of US $305 in the first year, yielding an ICUR of US-$4,013 per QALY gained (Table 3), demonstrating that the PAC project was cost saving.

### Clinical Implications for Health Policy

This study corroborated a previous series of Taiwan studies reporting that a PAC project for stroke patients improved quality of life and functional status at the time of hospital discharge (12, 27–29). In these studies, the largest improvements seemed to be achieved after 3 months of rehabilitation. In contrast, our repeated measures of multiple functional disabilities found although the non-PAC group performed better at baseline and first 6 weeks, PAC yielded significantly larger improvements in EQ-5D and all functional outcomes, except for FOIS, during weeks 7–12 of rehabilitation (Figure 2 and the difference in differences in Supplementary Table 2). And these scores did not significantly differ between the two groups from the end of the 12th week to 1 year. Moreover, the above improvements were accomplished under cost-saving condition.

Although inpatient stroke care by an organized multidisciplinary healthcare team could reduce mortality (30–32), treatment in an inpatient rehabilitation facility (IRF) was more expensive than treatment in a skilled nursing facility (SNF) (6, 33). Moreover, co-locating acute care and rehabilitation care for stroke in a district hospital was associated with reduced mortality and decreased length of hospital stay (34). As Table 3 presents, the average PAC cost of standard rehabilitative care in a single medical center in Taiwan where acute care and rehabilitative care are delivered in neurology ward, and rehabilitation ward, of tertiary-care hospital (US$3,785 per person) is higher than that of an intensive PAC project delivered in a secondary-care hospital (US$3,480 per person), where the improvement in functional status and reduced mortality were achieved at a lower cost. Additionally, the high maximum age in the PAC group (89 years old) suggests that advanced age alone should not be excluded from criteria for admission to a neurorehabilitation unit following acute stroke treatment (28).

### Limitations of This Study

The following limitations of this study must be acknowledged. First, being not a randomized trial, this study exercised rigorous matching to secure comparability at the expense of generalizability. Second, at baseline, the non-PAC group had higher average scores for EQ-5D, MMSE and BI compared to the PAC group. Therefore, the magnitude of functional improvements obtained by PAC may have been underestimated. Nonetheless, the potential bias would not change our conclusion that PAC seemed more cost-effective than non-PAC. Third, we did not include time to rehabilitation, distance to hospital, and costs (including productivity loss and out-of-pocket expenses) involved in the non-health care sector or societal perspective (35). Further research is required to explore stroke onset to rehabilitation, geographic location, and those costs. Fourth, this study did not collect data of detailed emergency intervention and co-medications, including thrombectomy, tissue plasminogen activator treatment, novel oral anticoagulants, antiplatelet drugs and medications for hypertension, diabetes and hyperlipidemia. Thus, their impacts on functional outcomes could not be ascertained. However, since all management of stroke patients must follow the guideline recommended by the Taiwan Stroke Society (36) to achieve the target values (37) and avoid rejection of reimbursement by Taiwan NHI, the likelihood of potential confounding by different emergency treatments and co-medications would not be too large. Fifth, repeated measures of functional outcomes were limited to 1 year. Further longitudinal follow-up studies are needed to assess long-term effect of a PAC project on functional outcomes, morbidity and mortality.

## Conclusions

The PAC group had lower direct medical costs and higher QALY gains compared to the non-PAC group during 1-year follow-up period. Thus, enrolling stroke patients into an organized multidisciplinary PAC project could significantly improve their functional status and saved medical costs. The improved effectiveness of PAC was corroborated by evidence of significant improvements in at least 4 functional outcomes during weeks 7-12 of rehabilitation. Further long-term research is required to validate its benefit on clinical outcomes, and should include survival and overall societal impact.

## Data Availability Statement

The raw data supporting the conclusions of this article will be made available by the authors, without undue reservation.

## Ethics Statement

The studies involving human participants were reviewed and approved by before study commencement, we obtained formal approval from the Institutional Review Board of Kaohsiung Medical University Hospital (KMUH-IRB-20140308). The patients/participants provided their written informed consent to participate in this study.

## Author Contributions

Y-CC, J-DW, and H-YS collated, analyzed, interpreted the data and wrote the manuscript, designed the study, provided statistical expertise, and wrote the first draft of the manuscript. Y-JY, H-FL, C-HL, H-HH, and K-WH collected the data. All authors contributed to the interpretation of the results, critical revision of the manuscript for important intellectual content, and have approved the final version of the manuscript. The corresponding author attests that all listed authors meet authorship criteria and that no authors who met the criteria for authorship have been omitted. All authors contributed to the article and approved the submitted version.

## Funding

This study was supported by the Ministry of Science and Technology (MOST 104-2410-H-037-006-SS2, MOST 106-2410-B-037−076, and MOST 108 2410 H 037 006 SS3) in Taiwan. The funder had no role in the design and conduct of the study; collection, management, analysis, and interpretation of the data; preparation, review, or approval of the manuscript; or decision to submit the manuscript for publication.

## Conflict of Interest

The authors declare that the research was conducted in the absence of any commercial or financial relationships that could be construed as a potential conflict of interest.

## Publisher's Note

All claims expressed in this article are solely those of the authors and do not necessarily represent those of their affiliated organizations, or those of the publisher, the editors and the reviewers. Any product that may be evaluated in this article, or claim that may be made by its manufacturer, is not guaranteed or endorsed by the publisher.

## Supplementary Material

Supplementary Figure 1 Comparison by LS-mean ± SE of each functional status measure between PAC and non-PAC groups, after controlling for baseline, week 6 and 12, and 1 year data and comparison of trend in each functional status measure between the groups. Utility_TW, Utility (Taiwan); Utility_UK, Utility (United Kingdom); MMSE, Mini-Mental State Examination; BI, Barthel index; IADL, Instrumental Activities of Daily Living; FOIS, Functional Oral Intake Scale; BBS, Berg Balance Scale; PAC, postacute care; LS-mean, least squares mean; SE, standard error. *P* values for trend in each functional status measure between the groups (120:120).

Supplementary Figure 2 Comparison by LS-mean ± SE for each functional status measure between PAC and non-PAC groups, after PSM of 116 vs. 69, at baseline, weeks 6 and 12, and 1 year, respectively, and trend for each functional status measure between the groups (62:62). Utility_TW, Utility (Taiwan); Utility_UK, Utility (United Kingdom); MMSE, Mini-Mental State Examination; BI, Barthel Index; IADL, Instrumental Activities of Daily Living; FOIS, Functional Oral Intake Scale; BBS, Berg Balance Scale; PAC, postacute care; LS-mean, least squares mean; SE, standard error. P values for trend for each functional status measure between the groups.

Supplementary Figure 3 Incremental cost effectiveness [postacute care (PAC) vs. non-PAC]. Scatter plots of incremental effectiveness (quality-adjusted life years) vs. incremental costs from 1,000 resamplings in the probabilistic sensitivity analysis with variation limited to cost and effectiveness assumptions and with transition-probabilities constant. This probabilistic sensitivity analysis demonstrated that PAC has a 100% probability of achieving cost savings relative to non-PAC in patients with stroke. Each plotted point is the result of an incremental cost divided by incremental quality-adjusted life years. The elliptic circle represents the 95% confidence interval.

Supplementary Figure 4 Cost-effectiveness acceptability curves for the probabilistic sensitivity analysis. Results from 1,000 resamplings in the probabilistic sensitivity analysis created synthetic populations of patients from the trial using bootstrapping. The lines represent the fraction of simulation iterations in which the postacute care (PAC) project was cost effective compared with standard rehabilitation (y-axis) at various levels of willingness to pay for quality-adjusted life years (QALYs) gains (x-axis).

Supplementary Figure 5 Comparison of LS-mean ± SE of each functional status measure between PAC and non-PAC groups, after 2:1 matching, at baseline, weeks 6 and 12, and 1 year and comparison of trend in each functional status measure between the groups. Utility_TW, utility (Taiwan); Utility_UK, utility (United Kingdom); MMSE, Mini-Mental State Examination; BI, Barthel Index; IADL, Instrumental Activities of Daily Living; FOIS, Functional Oral Intake Scale; BBS, Berg Balance Scale; PAC, postacute care; LS-mean, least squares mean; SE, standard error. P values for trend in each functional status measure between the PAC and non-PAC groups (164:82).
