# Supplementary material for: Cost Utility Analysis of Multidisciplinary Postacute Care for Stroke: A Prospective Six-Hospital Cohort Study
Source: Front Cardiovasc Med. 2022 Mar 30;9:826898. doi: 10.3389/fcvm.2022.826898 (PMC9007246; doi:10.3389/fcvm.2022.826898)
Supplement: Supplementary file 1 [file Table_1.DOC]

**eTABLE 1** Codes for stroke.

| **Conditions** | **ICD 9** | **ICD 10** |
| --- | --- | --- |
| Infraction | 433.00 | I65.1; I65.29 |
|  | 433.01 | I63.22 |
|  | 433.10 | I65.29 |
|  | 433.11 | I63.139 |
|  | 433.20 | I65.09 |
|  | 433.21 | I63.019; I63.119; I63.219 |
|  | 433.30 | I65.8 |
|  | 433.31 | I63.59 |
|  | 433.80 | I65.8 |
|  | 433.81 | I63.59 |
|  | 433.90 | I65.9 |
|  | 433.91 | I63.20 |
|  | 434.00 | I66.09; I66.19; I66.20 |
|  | 434.01 | I63.30 |
|  | 433.10 | I66.09; I66.19; I66.29; I66.9 |
|  | 434.11 | I63.40 |
|  | 434.90 | I66.9 |
|  | 434.91 | I63.50 |
|  | 436 | I67.89 |
|  |  | I63.9 |
| Hemorrhage | 430 | I60.9 |
|  | 431 | I61.9 |
