# Supplementary material for: Cost Utility Analysis of Multidisciplinary Postacute Care for Stroke: A Prospective Six-Hospital Cohort Study
Source: Front Cardiovasc Med. 2022 Mar 30;9:826898. doi: 10.3389/fcvm.2022.826898 (PMC9007246; doi:10.3389/fcvm.2022.826898)
Supplement: Supplementary file 2 [file Table_2.DOC]

**eTABLE 2** Comparison and trend for each functional status measure between PAC and non-PAC groups before and after rehabilitation after matching (120:120)

| Outcomes | |  | 6^th^ week - baseline | |  | 12^th^ week - 6^th^ week | |  | 1^st^ year - 12^th^ week | | *P* value  for trend^¶^ |
| --- | --- | --- | --- | --- | --- | --- | --- | --- | --- | --- | --- |
|  |  |  | LS-mean±SE  (T_1_-T_0_) | *P* value^†^ |  | LS-mean±SE  (T_2_-T_1_) | *P* value^†^ |  | LS-mean±SE  (T_3_-T_2_) | *P* value^†^ |  |
| Utility_TW | PAC |  | 0.00±0.02 | 0.080 |  | 0.16±0.02 | <0.001 |  | -0.04±0.02 | 0.630 | <0.001 |
|  | Non-PAC |  | 0.04±0.02 |  |  | -0.01±0.03 |  |  | -0.04±0.02 |  |  |
| Utility_UK | PAC |  | 0.02±0.03 | 0.370 |  | 0.21±0.03 | <0.001 |  | -0.04±0.02 | 0.660 | <0.001 |
|  | Non-PAC |  | 0.06±0.03 |  |  | -0.01±0.04 |  |  | -0.05±0.03 |  |  |
| MMSE | PAC |  | -0.07±0.53 | 0.550 |  | 1.94±0.64 | <0.001 |  | -0.34±0.52 | 0.280 | <0.001 |
|  | Non-PAC |  | 0.15±0.54 |  |  | -1.34±0.67 |  |  | -0.77±0.57 |  |  |
| BI | PAC |  | 3.13±1.71 | 0.020 |  | 13.24±1.91 | <0.001 |  | -3.33±1.69 | 0.060 | <0.001 |
|  | Non-PAC |  | 6.09±1.75 |  |  | 2.35±1.99 |  |  | -5.91±1.86 |  |  |
| IADL | PAC |  | -0.17±0.16 | 0.001 |  | 0.79±0.18 | <0.001 |  | -0.28±0.12 | 0.600 | <0.001 |
|  | Non-PAC |  | 0.38±0.17 |  |  | -0.12±0.19 |  |  | -0.32±0.13 |  |  |
| FOIS | PAC |  | 0.01±0.16 | 0.090 |  | -0.22±0.42 | 0.990 |  | -0.04±0.15 | 0.270 | 0.216 |
|  | Non-PAC |  | 0.23±0.16 |  |  | -0.22±0.45 |  |  | -0.17±0.16 |  |  |
| BBS | PAC |  | -0.74±1.08 | <0.001 |  | 11.41±1.37 | <0.001 |  | -2.45±0.97 | 0.150 | <0.001 |
|  | Non-PAC |  | 3.51±1.10 |  |  | -1.01±1.43 |  |  | -3.44±1.05 |  |  |

*Utility_TW, utility (Taiwan); Utility_UK, utility (United Kingdom); MMSE, mini-mental state examination; BI, Barthel index; IADL, instrumental activities of daily living; FOIS, functional oral intake scale; BBS, Berg balance scale; LS-mean, least squares mean; SE, standard error*.

*T_0_=Baseline; T_1_=6^th^ week; T_2_=12^th^ week; T_3_=1^st^ year.*

*^†^Comparison for each functional status measure between PAC and non-PAC groups on the differences of T_1_-T_0_, T_2_-T_1_ and T_3_-T_2_, respectively.*

*^¶^Trend for each functional status measure between PAC and non-PAC groups along the differences of T_1_-T_0_, T_2_-T_1_ and T_3_-T_2_ during the study period.*
