# Supplementary material for: Cost Utility Analysis of Multidisciplinary Postacute Care for Stroke: A Prospective Six-Hospital Cohort Study
Source: Front Cardiovasc Med. 2022 Mar 30;9:826898. doi: 10.3389/fcvm.2022.826898 (PMC9007246; doi:10.3389/fcvm.2022.826898)
Supplement: Supplementary file 3 [file Table_3.DOC]

**eTABLE 3** Comparison and trend for differences of each functional status measure between PAC and non-PAC groups after matching (120:120)

| Outcome | | 6^th^ week - baseline | | |  | 12^th^ week - baseline | | |  | 1^st^ year - baseline | | | *P* value  for trend^¶^ |
| --- | --- | --- | --- | --- | --- | --- | --- | --- | --- | --- | --- | --- | --- |
|  |  | LS-mean±SE  (T_1_-T_0_) | *P* value^†^ |  |  | LS-mean±SE  (T_2_-T_0_) | *P* value^†^ |  |  | LS-mean±SE  (T_3_-T_0_) | *P* value^†^ |  |  |
| Utility_TW | PAC | 0.01±0.04 | 0.060 |  |  | 0.21±0.04 | <0.001 |  |  | 0.21±0.04 | <0.001 |  | <0.001 |
|  | Non-PAC | 0.05±0.04 |  |  |  | 0.08±0.04 |  |  |  | 0.07±0.04 |  |  |  |
| Utility_UK | PAC | 0.04±0.06 | 0.350 |  |  | 0.29±0.06 | <0.001 |  |  | 0.29±0.06 | <0.001 |  | <0.001 |
|  | Non-PAC | 0.07±0.06 |  |  |  | 0.10±0.07 |  |  |  | 0.10±0.07 |  |  |  |
| MMSE | PAC | 0.06±0.71 | 0.500 |  |  | 2.45±0.83 | <0.001 |  |  | 2.52±0.88 | <0.001 |  | <0.001 |
|  | Non-PAC | 0.31±0.72 |  |  |  | -0.59±0.85 |  |  |  | -1.00±0.91 |  |  |  |
| BI | PAC | 6.53±2.35 | 0.010 |  |  | 22.80±2.64 | 0.001 |  |  | 22.75±2.77 | 0.001 |  | <0.001 |
|  | Non-PAC | 9.92±2.41 |  |  |  | 15.60±2.70 |  |  |  | 13.2±2.91 |  |  |  |
| IADL | PAC | -0.06±0.29 | 0.001 |  |  | 1.02±0.30 | 0.170 |  |  | 1.05±0.30 | 0.170 |  | 0.001 |
|  | Non-PAC | 0.54±0.30 |  |  |  | 0.75±0.31 |  |  |  | 0.76±0.31 |  |  |  |
| FOIS | PAC | 0.40±0.24 | 0.060 |  |  | 0.23±0.46 | 0.720 |  |  | 0.23±0.46 | 0.870 |  | 0.640 |
|  | Non-PAC | 0.65±0.25 |  |  |  | 0.43±0.47 |  |  |  | 0.32±0.48 |  |  |  |
| BBS | PAC | -0.73±1.70 | <0.001 |  |  | 13.11±1.94 | <0.001 |  |  | 13.28±1.97 | <0.001 |  | <0.001 |
|  | Non-PAC | 3.62±1.74 |  |  |  | 5.24±1.98 |  |  |  | 4.60±2.04 |  |  |  |

*Utility_TW, utility (Taiwan); Utility_UK, utility (United Kingdom); MMSE, mini-mental state examination; BI, Barthel index; IADL, instrumental activities of daily living; FOIS, functional oral intake scale; BBS, Berg balance scale; LS-mean, least squares mean; SE, standard error.*

*T_0_=Baseline; T_1_=6^th^ week; T_2_=12^th^ week; T_3_=1^st^ year.
^†^Comparison for differences of each functional status measure between PAC and non-PAC groups by controlling the baseline via T_1_-T_0_, T_2_-T_0_ and T_3_-T_0_, respectively.*

*^¶^Trend for differences of each functional status measure between PAC and non-PAC groups after controlling the baseline at 6^th^ week, 12^th^ week and 1^st^ year, respectively.*
