# Supplementary material for: Cost Utility Analysis of Multidisciplinary Postacute Care for Stroke: A Prospective Six-Hospital Cohort Study
Source: Front Cardiovasc Med. 2022 Mar 30;9:826898. doi: 10.3389/fcvm.2022.826898 (PMC9007246; doi:10.3389/fcvm.2022.826898)
Supplement: Supplementary file 4 [file Table_4.DOC]

**eTABLE 4** Comparison and trend of each functional status measure between PAC and non-PAC groups before and after rehabilitation after matching (120:120)

| Outcomes | | Baseline | |  | 6^th^ week after rehabilitation | |  | 12^th^ week after rehabilitation | |  | 1^st^ year after rehabilitation | |
| --- | --- | --- | --- | --- | --- | --- | --- | --- | --- | --- | --- | --- |
|  |  | Mean±SE | *P* value^†^ |  | Mean±SE | ES (T_1_-T_0_) |  | Mean±SE | ES (T_2_-T_1_) |  | Mean±SE | ES (T_3_-T_2_) |
| Utility_TW | PAC | 0.40±0.02 | <0.0001 |  | 0.44±0.02 | 0.21 |  | 0.64±0.02 | 0.86 |  | 0.63±0.02 | -0.02 |
|  | Non-PAC | 0.57±0.02 |  |  | 0.65±0.02 | 0.33 |  | 0.68±0.02 | 0.11 |  | 0.74±0.03 | 0.22 |
| Utility_UK | PAC | 0.19±0.03 | <0.0001 |  | 0.26±0.03 | 0.21 |  | 0.50±0.03 | 0.73 |  | 0.50±0.03 | -0.02 |
|  | Non-PAC | 0.42±0.03 |  |  | 0.53±0.03 | 0.30 |  | 0.56±0.03 | 0.09 |  | 0.63±0.04 | 0.20 |
| MMSE | PAC | 19.63±0.75 | 0.040 |  | 19.97±0.76 | 0.04 |  | 22.35±0.70 | 0.30 |  | 22.37±0.69 | 0.01 |
|  | Non-PAC | 21.81±0.76 |  |  | 22.40±0.78 | 0.07 |  | 21.79±0.84 | 0.07 |  | 23.39±0.95 | 0.19 |
| BI | PAC | 40.42±1.92 | 0.001 |  | 46.36±2.00 | 0.28 |  | 62.65±2.30 | 0.70 |  | 62.28±2.34 | -0.01 |
|  | Non-PAC | 50.00±2.23 |  |  | 60.27±2.31 | 0.42 |  | 65.83±2.53 | 0.22 |  | 70.14±3.01 | 0.17 |
| IADL | PAC | 1.20±0.10 | 0.090 |  | 1.32±0.10 | 0.11 |  | 2.39±0.14 | 0.79 |  | 2.37±0.14 | -0.01 |
|  | Non-PAC | 0.93±0.12 |  |  | 1.65±0.20 | 0.41 |  | 1.90±0.19 | 0.13 |  | 2.04±0.24 | 0.07 |
| FOIS | PAC | 6.61±0.55 | 0.470 |  | 6.66±0.56 | 0.01 |  | 6.50±0.11 | -0.04 |  | 6.51±0.11 | 0.01 |
|  | Non-PAC | 6.19±0.17 |  |  | 6.58±0.13 | 0.24 |  | 6.40±0.16 | -0.11 |  | 6.65±0.15 | 0.16 |
| BBS | PAC | 15.53±1.41 | 0.190 |  | 17.22±1.51 | 0.11 |  | 30.92±1.60 | 0.81 |  | 31.11±1.63 | 0.01 |
|  | Non-PAC | 18.23±1.47 |  |  | 24.45±1.64 | 0.37 |  | 26.78±1.70 | 0.13 |  | 30.53±2.14 | 0.21 |

*Utility_TW, utility (Taiwan); Utility_UK, utility (United Kingdom); MMSE, mini-mental state examination; BI, Barthel index; IADL, instrumental activities of daily living; FOIS, functional oral intake scale; BBS, Berg balance scale; SE, standard error; ES, effect size.*

*T_0_=Baseline; T_1_=6^th^ week; T_2_=12^th^ week; T_3_=1^st^ year.*

*^†^P value is calculated by t-test.*
