# Supplementary material for: Cost Utility Analysis of Multidisciplinary Postacute Care for Stroke: A Prospective Six-Hospital Cohort Study
Source: Front Cardiovasc Med. 2022 Mar 30;9:826898. doi: 10.3389/fcvm.2022.826898 (PMC9007246; doi:10.3389/fcvm.2022.826898)
Supplement: Supplementary file 5 [file Table_5.DOC]

**eTABLE 5** Distributions of patient characteristics at one year and after re-matching on demographics and PS (propensity scores)

| Variables | | Before re-matching | | | After re-matching | | |
| --- | --- | --- | --- | --- | --- | --- | --- |
|  |  | PAC  (*n*=1,16) | Non-PAC  (*n*=69) | *P* value | PAC  (*n*=62) | Non-PAC  (*n*=62) | *P* value |
| Cerebrovascular accident  (CVA) year of diagnosis | 2015 2016 2017 | 11 (9.48%)  64 (55.17%)  41 (35.34%) | 5 (7.25%)  46 (66.67%)  18 (26.09%) | 0.305 | 9 (14.52%)  36 (58.06%)  17 (27.42%) | 5 (8.06%)  40 (64.52%)  17 (27.42%) | 0.508 |
| Age, years^†^ |  | 67.31±12.25 | 66.19±11.75 | 0.542 | 66.89±12.17 | 67.13±10.88 | 0.907 |
| Number of stroke patients (%) | | 116 (100%) | 69 (100%) |  | 62(100%) | 62(100%) |  |
| Gender (Male %) | | 78 (67.24%) | 47 (68.12%) | 0.902 | 43 (69.35%) | 42 (67.74%) | 0.847 |
| Nasogastric tube No. (%) | | 10 (8.62%) | 5 (7.25%) | 0.741 | 55 (88.71%) | 57 (91.94%) | 0.544 |
| Foley No. (%) | | 8 (6.9%) | 3 (4.35%) | 0.478 | 57 (91.94%) | 59 (95.16%) | 0.465 |
| Education, years^†^ | | 8.86±1.74 | 9.22±5.04 | 0.489 | 9.08±1.71 | 8.94±4.93 | 0.827 |
| BMI, kg/m^2†^ | | 24.22±2.3 | 24.69±3.46 | 0.274 | 24.25±2.16 | 24.3±3.22 | 0.920 |
| Stroke type: Ischemic (%) | | 107 (92.24%) | 63 (91.3%) | 0.821 | 55 (88.71%) | 57 (91.94%) | 0.544 |
| Hemorrhagic (%) | | 9 (7.76%) | 6 (8.7%) |  | 7 (11.29%) | 5 (8.06%) |  |
| Hypertension No. (%) | | 75 (64.66%) | 47 (68.12%) | 0.631 | 18 (29.03%) | 18 (29.03%) | 1 |
| Diabetes mellitus No. (%) | | 45 (38.79%) | 27 (39.13%) | 0.964 | 44 (70.97%) | 40 (64.52%) | 0.442 |
| Hyperlipidemia No. (%) | | 36 (31.03%) | 19 (27.54%) | 0.615 | 44 (70.97%) | 44 (70.97%) | 1 |
| Atrial fibrillation (Yes %) | | 8 (6.9%) | 5 (7.25%) | 0.928 | 59 (95.16%) | 58 (93.55%) | 0.697 |
| Previous stroke (Yes %) | | 17 (14.66%) | 9 (13.04%) | 0.760 | 9 (14.52%) | 9 (14.52%) | 1 |

*PAC, post-acute care;* BMI, Body mass index.

*^†^Values are expressed as mean ± standard deviation.*
