# Supplementary material for: Cost Utility Analysis of Multidisciplinary Postacute Care for Stroke: A Prospective Six-Hospital Cohort Study
Source: Front Cardiovasc Med. 2022 Mar 30;9:826898. doi: 10.3389/fcvm.2022.826898 (PMC9007246; doi:10.3389/fcvm.2022.826898)
Supplement: Supplementary file 6 [file Table_6.DOC]

**eTABLE 6** Comparison and trend for each functional status measure between PAC and non-PAC groups after re-matching (62:62)

| Outcome | | Baseline | | |  | 6^th^ week after rehabilitation | | |  | 12^th^ week after rehabilitation | | |  | 1^st^ year after rehabilitation | | | *P* value  for trend^¶^ |
| --- | --- | --- | --- | --- | --- | --- | --- | --- | --- | --- | --- | --- | --- | --- | --- | --- | --- |
|  |  | LS-mean±SE | | *P* value^†^ |  | LS-mean±SE | *P* value^†^ |  |  | LS-mean±SE | *P* value^†^ |  |  | LS-mean±SE | *P* value^†^ |  |  |
| Utility_  TW | PAC | 0.27±0.07 | <0.001 | |  | 0.29±0.07 | <0.001 |  |  | 0.50±0.07 | 0.040 |  |  | 0.50±0.07 | 0.050 |  | < 0.001 |
|  | Non-PAC | 0.48±0.07 |  | |  | 0.59±0.07 |  |  |  | 0.59±0.07 |  |  |  | 0.59±0.07 |  |  |  |
| Utility_  UK | PAC | -0.02±0.10 | <0.001 | |  | 0.02±0.10 | <0.001 |  |  | 0.28±0.10 | 0.060 |  |  | 0.28±0.10 | 0.070 |  | < 0.001 |
|  | Non-PAC | 0.26±0.10 |  | |  | 0.39±0.11 |  |  |  | 0.40±0.11 |  |  |  | 0.39±0.11 |  |  |  |
| MMSE | PAC | 11.52±2.31 | 0.040 | |  | 11.88±2.31 | 0.020 |  |  | 14.14±2.29 | 0.290 |  |  | 14.22±2.28 | 0.530 |  | 0.020 |
|  | Non-PAC | 14.21±2.35 |  | |  | 15.14±2.36 |  |  |  | 15.47±2.34 |  |  |  | 14.97±2.33 |  |  |  |
| BI | PAC | 10.04±5.79 | <0.001 | |  | 16.22±5.80 | <0.001 |  |  | 33.27±5.94 | 0.004 |  |  | 33.27±6.02 | 0.040 |  | < 0.001 |
|  | Non-PAC | 27.23±5.91 |  | |  | 37.21±5.93 |  |  |  | 43.98±6.07 |  |  |  | 41.27±6.13 |  |  |  |
| IADL | PAC | 0.47±0.39 | 0.970 | |  | 0.63±0.40 | 0.200 |  |  | 1.67±0.41 | 0.310 |  |  | 1.67±0.41 | 0.290 |  | 0.040 |
|  | Non-PAC | 0.48±0.40 |  | |  | 0.94±0.41 |  |  |  | 1.39±0.42 |  |  |  | 1.37±0.42 |  |  |  |
| FOIS | PAC | 4.43±0.32 | 0.020 | |  | 4.51±0.32 | 0.003 |  |  | 4.97±0.31 | 0.440 |  |  | 4.97±0.32 | 0.930 |  | 0.020 |
|  | Non-PAC | 4.89±0.32 |  | |  | 5.14±0.32 |  |  |  | 5.11±0.32 |  |  |  | 4.99±0.33 |  |  |  |
| BBS | PAC | 0.93±4.42 | 0.002 | |  | 2.70±4.44 | <0.001 |  |  | 16.10±4.49 | 0.530 |  |  | 16.27±4.51 | 0.790 |  | < 0.001 |
|  | Non-PAC | 8.15±4.51 |  | |  | 14.87±4.54 |  |  |  | 17.72±4.58 |  |  |  | 16.96±4.60 |  |  |  |

*Utility_TW, utility(Taiwan); Utility_UK, utility(United Kingdom); MMSE, mini-mental state examination; BI, Barthel index; IADL, instrumental activities of daily living; FOIS, functional oral intake scale; BBS, Berg balance scale; LS-mean, least squares mean; SE, standard error.*

*T_0_=Baseline; T_1_=6^th^ week; T_2_=12^th^ week; T_3_=1^st^ year.*

*^†^Comparison for each functional status measure between PAC and non-PAC groups at baseline, 6^th^ week, 12^th^ week and 1^st^ year, respectively.*

*^¶^Trend for each functional status measure between PAC and non-PAC groups during the study period.*
