# Supplementary material for: Cost Utility Analysis of Multidisciplinary Postacute Care for Stroke: A Prospective Six-Hospital Cohort Study
Source: Front Cardiovasc Med. 2022 Mar 30;9:826898. doi: 10.3389/fcvm.2022.826898 (PMC9007246; doi:10.3389/fcvm.2022.826898)
Supplement: Supplementary file 7 [file Table_7.DOC]

**eTABLE 7 Comparison of functional status trends between PAC and non-PAC groups after matching (164:82)**

| Outcomes | | Baseline  (T_0_) | |  | 6^th^ week after rehabilitation  (T_1_) | |  | 12^th^ week after rehabilitation  (T_2_) | |  | 1^st^ year after rehabilitation  (T_3_) | | *P* value  for trend^¶^ |
| --- | --- | --- | --- | --- | --- | --- | --- | --- | --- | --- | --- | --- | --- |
|  |  | **LS-mean±SE** | *P* value^†^ |  | **LS-mean±SE** | *P* value^†^ |  | **LS-mean±SE** | *P* value^†^ |  | **LS-mean±SE** | *P* value^†^ |  |
| Utility_  TW | PAC | 0.14±0.07 | <0.001 |  | 0.18±0.07 | <0.001 |  | 0.37±0.07 | 0.411 |  | 0.37±0.07 | 0.227 | <0.001 |
|  | Non-PAC | 0.29±0.07 |  |  | 0.38±0.07 |  |  | 0.4±0.08 |  |  | 0.41±0.08 |  |  |
| Utility_UK | PAC | -0.2±0.11 | <0.001 |  | -0.15±0.11 | <0.001 |  | 0.09±0.11 | 0.402 |  | 0.09±0.11 | 0.245 | <0.001 |
|  | Non-PAC | 0.00±0.11 |  |  | 0.10±0.11 |  |  | 0.12±0.11 |  |  | 0.14±0.11 |  |  |
| MMSE | PAC | 12.77±2.79 | 0.051 |  | 13.23±2.79 | 0.040 |  | 15.43±2.8 | 0.221 |  | 15.58±2.79 | 0.072 | <0.001 |
|  | Non-PAC | 14.79±2.85 |  |  | 15.33±2.85 |  |  | 14.14±2.86 |  |  | 13.7±2.86 |  |  |
| BI | PAC | 3.33±7.98 | 0.015 |  | 8.93±7.99 | <0.001 |  | 26.88±8.01 | 0.929 |  | 27.24±8.02 | 0.341 | <0.001 |
|  | Non-PAC | 10.18±8.13 |  |  | 19.83±8.15 |  |  | 26.60±8.21 |  |  | 24.11±8.27 |  |  |
| IADL | PAC | 0.47±0.44 | 0.002 |  | 0.54±0.45 | 0.263 |  | 1.67±0.46 | 0.007 |  | 1.71±0.46 | 0.005 | <0.001 |
|  | Non-PAC | 0.00±0.45 |  |  | 0.73±0.46 |  |  | 1.04±0.47 |  |  | 1.04±0.48 |  |  |
| FOIS | PAC | 3.20±0.53 | 0.662 |  | 3.25±0.53 | 0.781 |  | 3.23±0.42 | 0.214 |  | 3.23±0.42 | 0.070 | 0.779 |
|  | Non-PAC | 2.94±0.62 |  |  | 3.09±0.62 |  |  | 3.04±0.42 |  |  | 2.93±0.43 |  |  |
| BBS | PAC | -4.10±5.61 | 0.432 |  | -2.69±5.62 | 0.005 |  | 13.07±5.63 | <0.001 |  | 13.24±5.63 | <0.001 | <0.001 |
|  | Non-PAC | -2.48±5.73 |  |  | 3.37±5.75 |  |  | 4.84±5.76 |  |  | 4.34±5.78 |  |  |

*PAC, post-acute care; Utility_TW, utility (Taiwan); Utility_UK, utility (United Kingdom); MMSE, Mini-Mental State Examination; BI, Barthel index; IADL, Instrumental Activities of Daily Living; FOIS, Functional Oral Intake Scale; BBS, Berg Balance Scale; LS-mean, least squares mean; SE, standard error;*

*^†^Each functional status measure was compared between the PAC and non-PAC groups at baseline and after 6, 12, and 52 weeks.*

*^¶^Trends in differences between PAC and non-PAC groups for each functional status measure during the study period.*
