# Supplementary material for: Cost Utility Analysis of Multidisciplinary Postacute Care for Stroke: A Prospective Six-Hospital Cohort Study
Source: Front Cardiovasc Med. 2022 Mar 30;9:826898. doi: 10.3389/fcvm.2022.826898 (PMC9007246; doi:10.3389/fcvm.2022.826898)
Supplement: Supplementary file 8 [file Table_8.DOC]

**eTABLE 8 Cost-utility analysis of PAC and non-PAC groups within 1 year after stroke rehabilitation (164:82)**

|  | PAC group  (*n*=164)  mean ± SD | Non-PAC group  (*n*=82)  mean ± SD (%) | Incremental difference  (PAC – non-PAC)^¶^  mean ± SD (%) |
| --- | --- | --- | --- |
| *Baseline* |  |  |  |
| Utility score | 0.44 ± 0.20 | 0.59 ± 0.23 | - (0.15 ± 0.21)^c^ |
| *1 year after stroke rehabilitation* |  |  |  |
| NHI total direct medical cost^†^ | 3,396 ± 1,695 | 3,600 ± 4,025 | - (205 ± 2,702) |
| Utility score | 0.67 ± 0.24 | 0.77 ± 0.26 | - (0.10 ± 0.24)^#^ |
| QALYs gained^§^ | 0.1948 | 0.1052 | 0.0896 |
| ICUR (PAC – non-PAC) | dominant |  | -2,288 |

*PAC, post-acute care; mean, arithmetic mean; SD, standard deviation; NHI, national health insurance; QALYs, quality adjusted life years; ICUR, incremental cost-utility ratio.*

*^†^Mean direct cost for the PAC group. Per diem reimbursement packages received by hospitals varied by intensity of rehabilitation, e.g., per diem reimbursement for high-intensity rehabilitative care was the maximal packaged reimbursement of NT$3,587; per diem reimbursement for usual rehabilitative care was NT$2,411 (2019 exchange rate, NT $30.5=US $1). Reimbursement included fees for physician, ward service, nursing, laboratory, rehabilitation therapy, and medication/pharmacy service fee, etc.*

*^§^Area under the curve with control for baseline utility.*

*^¶^P<0.001 for independent t test of the two groups.*

*^#^P=0.01 for independent t test of the two groups.*
