# Supplementary material for: Cost Utility Analysis of Multidisciplinary Postacute Care for Stroke: A Prospective Six-Hospital Cohort Study
Source: Front Cardiovasc Med. 2022 Mar 30;9:826898. doi: 10.3389/fcvm.2022.826898 (PMC9007246; doi:10.3389/fcvm.2022.826898)
Supplement: Supplementary file 9 [file Data_Sheet_1.PDF]

Supplementary document that briefly summarizes the Post-Acute Care-Cerebrovascular Diseases (PAC-CVD) project in Taiwan

When Taiwan launched a PAC-CVD project in 2014 to contain PAC expenditures, an official document of its pilot study was published. Figure 1 shows the flow diagram of PAC-CVD; Table 1 describes features of PAC project vs. non-PAC care. We condense 40 pages of Chinese contents as follows:

Background:

Literature review shows that readmission and prolonged length of stay for stroke patients are common, and it is one of top ten diseases, among which post-acute care is required in the United States (1, 2). 62.6-74.5% received post-acute care within 30 days after discharge (3). Buntin et al. have reported that among stroke patients, use of an inpatient rehabilitation facility (IRF) reduced mortality by 2.7% ($P < 0.001$) compared with returning home, whereas there was no difference in mortality between using a skilled nursing facility (SNF) and returning home (4).

In Taiwan, among 2,358 stroke subjects eligible for analysis, 10.4% had prolonged hospital stay, but they accounted for 38.9% of the total person-hospital days and 47.8% of the total in-hospital medical expenses (5, 6). The hospital readmission rate within 6 months after index hospitalization was 46.5% in Taiwan (7), while in United States, it was significantly lower in the intervention groups (34%) than in the control group (44%, $P = 0.028$) (8), and in Australia, it was 27% in conventional care and 36% in home–based scheme (9). Bravata et al. reported that 53.3% had died or been readmitted at least once during the first year after discharge, and 14.3 survived admission-free for 5 years (10). One study of Taiwan estimated that 10.11%-23.13% of stroke patients may need PAC. PAC days accounted for 16.94-44.68% of the total length of stay. The hospital days per stroke patient may be reduced by 2.06-8.17 days, and hospital bed occupancy may be reduced by 306-1,210 beds after PAC (11).

A 4-week community-hospital-based interdisciplinary PAC project significantly improved functional recovery and reduced 12-month mortality by 62% for older post-acute patients (12). A 4-week inpatient physical re-ablement project conducted by an interdisciplinary geriatric team in a community hospital can successfully improve the physical and mental function, mood, ambulation, and nutritional conditions of post-acute patients (13). The acute ward readmission rate within 14 days and 30 days of PAC care was 6.3% and 12.9%, respectively. The latter was lower than that reported in the general population (19.6%) and in chronic rehabilitation units (17.9%). In addition, the length of hospital stay is shorter in those who received PAC than those who did not (14). Post-acute care seems to be a service model deserving investigation.

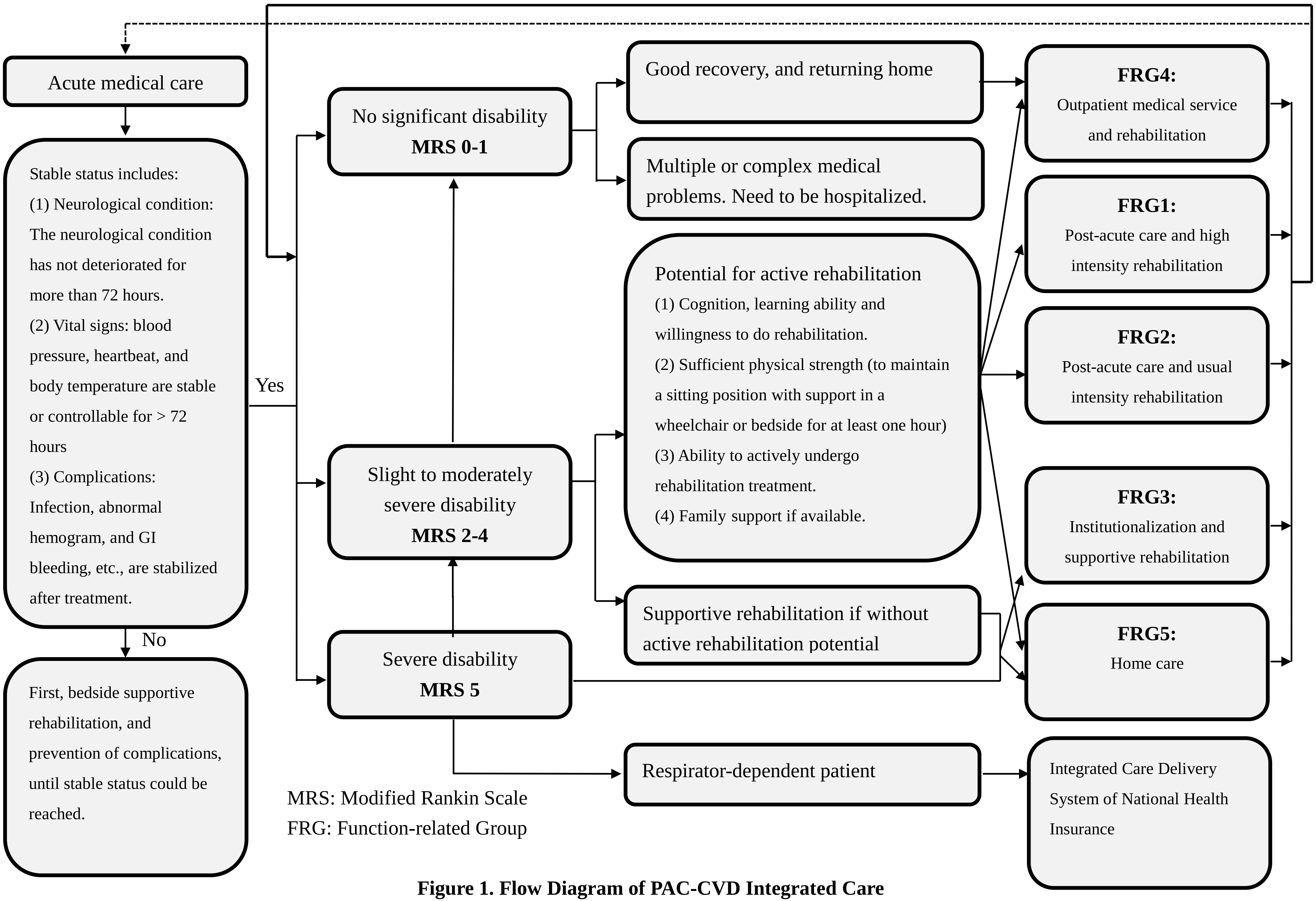

Figure 1. Flow Diagram of PAC-CVD Integrated Care

Aims:

1. To establish a post-acute care model to improve the quality of post-acute care in Taiwan.
2. To establish a vertically integrated transfer system for acute, post-acute and chronic phases to enhance the continuity of care for stroke patients.
3. To establish an organized multidisciplinary approach to securing effective and affordable post-acute care, and providing timely and integrated care for patients in the golden period to restore function, reduce disability, and prevent subsequent re-hospitalization.

Potential candidates include those who have:
(1) cognitive skills, learning ability and willingness.
(2) sufficient physical strength and resilience: ability to maintain a sitting posture for at least one hour in a wheelchair or bedside under support.
(3) capacity to actively participate in rehabilitation treatment plans.
(4) sufficient family support if available.

Rehabilitation project for PAC-CVD:
The multidisciplinary PAC stroke team consisted of neurologists, physiatrists, physiotherapists, occupational therapists, speech therapists and nurses. The PAC rehabilitation project was prescribed by the physiatrist, and it consisted of a complex project of universal activities that were performed three to five sessions per day. One hour of physical therapy, occupational therapy or speech or swallowing therapy was carried out at each session.
Physical therapy:
1. In the early stage of the disease or in the non-active rehabilitation period, including joint mobility exercises, stretching exercises, rehabilitation machinery or artificial assisted therapy, tilt table training, massage, joint mobilization, bed exercises, cognitive training, and breathing to expectorate phlegm, etc., to assist patients in recovering and preventing secondary injury or disability; Bobath neurodevelopmental treatment or proprioceptor neuromuscular induction and promotion technology to promote sensory-motor integration and induce patient motor recovery.
2. Active rehabilitation period, including bed and mat exercise, movement relearning, task-oriented training, early load-bearing suspension treadmill training method, dual-task training, rehabilitation machinery or artificial assisted therapy, virtual reality training, and use of assistive devices for action, etc., to achieve the training goals of enhancing muscle strength, muscle endurance, posture control, balance, gait, coordination and cardiorespiratory endurance, and at the same time have cognitive

training functions. Bobath neurodevelopmental treatment or proprioceptor neuromuscular induction and promotion technology promotes sensory-motor integration and recovery induction of patient movement.

3. Upper extremity exercise therapy, using functional electrical stimulation, limited therapy, Bobath neurodevelopmental therapy, proprioceptor neuromuscular induction and promotion technology, mechanical assisted therapy and virtual reality training, etc.

4. In a few cases, physical factors are used as adjuvant therapy: alternating hot and cold stimulation is used to reduce muscle tone, or cold therapy/heat therapy/electric therapy/hydrotherapy is used to treat shoulder pain.

(2) Occupational therapy content: Occupational therapy evaluation and plan formulation

prevention of secondary injury and disability prevention. activities of daily living function training (therapeutic activity design), activity therapy, social function training. Community/society participation training, psychosocial adaptation.

Group therapy, recreational sports functional training, movement disorders: contemporary corrective therapy (limited induction therapy, two-limb training, mirror therapy; mechanical assisted therapy), task-oriented therapy, improvement of personal factors related to movement, treatment of secondary damage, strengthening of movement recovery, use of activities to improve movement, perception, cognitive skills, use of activities to improve lower limb function and balance; use of activities to improve upper limb movement skills.

Sensory, perception, and cognitive impairment: visual perception correction and adaptive therapy, neglect syndrome correction therapy and multi-situation therapy, apraxia adaptive therapy and compensation strategy training, attention deficit correction and adaptive therapy, memory impairment therapeutic and adaptive therapy, executive function correction and multi-situation therapy. Assistive tools, auxiliary wood and home environment renovation.

(3) Speech therapy content: including electrical stimulation of swallowing or language function, oral function training, pharyngeal muscle function training, indirect swallowing exercise training, swallowing reflex induction training, swallowing compensation skills training, safe swallowing skills training, safe eating and drinking training, vocalization training, vocal cord function training, speech clarity training, assessment and training of communication aid, auditory comprehension training, oral expression training, language cognitive training, reading comprehension training, written language training, non-verbal communication skills training, language

communication and dysphagia consultation, all of which are reimbursed by National Health Insurance.

The closing or discharge regulations state that patients show: (I) functional improvement and the ability to practice rehabilitation at home, (II) no functional improvement based on two consecutive functional evaluations, (III) no potential for functional improvement based on stroke-team evaluation, (IV) patients have completed intensified rehabilitation of 12 weeks, (V) patients quit this project, or (VI) patients died (15).

Table 1. Features of PAC Project vs. Non PAC care

Rehabilitation	Multidisciplinary project<br>(PAC project)	Usual care<br>(non-PAC care)
Setting	Regional/district hospital	Medical center
Team member	Organized team of neurologists, physiatrists, physiotherapists, occupational therapists, speech therapists and nurses.	Qualified PAC professionals in non-PAC care, but not organized into an integrated team to serve post-acute stroke patients.
Reimbursement	Per diem	Fee for service
Intensity of rehabilitation	High intensity<br>Function-related group 1 (FRG1): NT$3,486 per day if 3-5 sessions a day in the 12 weeks; physical therapy:1-2 sessions per week day 30-60 minutes each session; 1 session per weekend, 30-60 minutes each session<br>Occupational therapy: 1-2 sessions per weekday, 30-60 minutes each session; 1 session per weekend;<br>Speech therapy: at least 5 sessions a week, depending on how a patient can communicate and/or swallow.	Usual intensity<br>Function-related group 1 (FRG1): NT$600 per session; 1 session per week day for physical therapy, occupational therapy and/or speech therapy, respectively; 30-60 minutes each session. From rehabilitation day to the end of 28 days, the longest duration allowed for hospitalization of acute stroke, or 40 days if patient is initially admitted into intensive care unit. The limitation of length of stay to 28 or 40 days seems to be a consensus in Taiwan.<br>Then rehabilitation is shifted into the context of outpatient service.

Intensity of rehabilitation	Usual intensity<br>Function-related group 2 (FRG2): NT$2,310 per day in the 12 weeks if 1-3 sessions a day; physical therapy:1 session per week day<br>30-60 minutes each session; 1 session per weekend, 30-60 minutes a session<br>Occupational therapy: 1-2 session per weekday, 30-60 minutes a session;<br>Speech therapy: at least 3-4 sessions a week, depending on how a patient can communicate and/or swallow.	Usual intensity and less frequency<br>Function-related group 2 (FRG2): NT$480 per session; a session per week day for physical therapy, occupational therapy and/or speech therapy, respectively; 30-60 minutes each session. From rehabilitation day to discharge day.<br>Then rehabilitation is shifted into the context of outpatient service.
Initial assessment fee:	Initial assessment fee:<br>NT$1,000.	No such incentive in non-PAC care
Periodic assessment fee	Periodic assessment fee,<br>once every three week, and its documentation is ordered to upload.	No such incentive in non-PAC care
Interhospital physicians discussion	Inter-hospital physicians discussion (vertical link between medical center, and regional/district, physicians) on progress of patient rehabilitation:<br>NT$1.000, once a week in the first three weeks.	Stroke patients stay in medical center until discharge.<br>Intra-hospital physicians discussion routinely in transfer of patient to rehabilitation ward. No incentive is given
Fee for home visit	Home visit by team member of PAC project,<br>Once before discharge and once after discharge,<br>At least 30minutes a visit: NT$1,000; if two or more team member visit together, fee is uptitrated to NT$1,500.	No such incentive in non-PAC care

Fee NT$60,000. for quality collaboration of vertically-linked hospitals	Fee NT$60,000. for quality collaboration of vertically-linked hospitals if the following criteria are met: 1. Inter-hospital team meetings should be held to set, adjust and reach goal. 2. Seamless transfer of patients should be coordinated. 3. Education project of multidisciplinary team should be scheduled and carried out. 4. Quality of PAC should be enhanced continuously. 5. Quality indicators of hospital team should be summarized and reviewed once half a year to provide quality of care. All above activities should be reported in the progress document every six months.	No such incentive in non-PAC care

References:

1. Department of Health and Human Services Centers for Medicare and Medicaid Services. Medicare Project; Proposed & Final Changes to the Hospital Inpatient Prospective Payment Systems and Fiscal Year 2009 Rates; Federal Register. http://wwwcmsgov/Medicare/Medicare-Fee-for-Service-Payment/AcuteInpatientPPS/IPPS-Regulations-and-Notices-Items/CMS1227598html Accessed August 18, 2009
2. Hoverman C, Shugarman LR, Saliba D, et al. Use of postacute care by nursing home residents hospitalized for stroke or hip fracture: how prevalent and to what end? *J Am Geriatr Soc.* (2008) 56(8):1490-6.
3. Kane RL, Lin WC, Blewett LA. Geographic variation in the use of post-acute care. *Health Serv Res.* (2002) 37(3):667-82.
4. Buntin MB, Colla CH, Deb P, et al. Medicare spending and outcomes after postacute care for stroke and hip fracture. *Med Care.* (2010) 48(9):776-84.
5. Tseng MC, Lin HJ. Readmission after hospitalization for stroke in Taiwan: results from a national sample. *J Neurol Sci.* (2009) 284(1-2):52-5.
6. Lee HC, Chang KC, Lan CF, et al. Factors associated with prolonged hospital stay for acute stroke in Taiwan. *Acta Neurol Taiwan.* (2008) 17(1):17-25.
7. Guey-Ing Day S-CW, Shin-Chung Huang. Use of Post-Hospital Care to Disabled Stroke Patients in Taiwan: A National Health Insurance Experience. *Disabil Health J.* (2009) 2(1):e1-e2.
8. Andersen HE, Schultz-Larsen K, Kreiner S, et al. Can readmission after stroke be prevented? Results of a randomized clinical study: a postdischarge follow-up service for stroke survivors. *Stroke.* (2000) 31(5):1038-45.
9. Anderson C, Rubenach S, Mhurchu CN, et al. Home or hospital for stroke rehabilitation? results of a randomized controlled trial : I: health outcomes at 6 months. *Stroke.* (2000) 31(5):1024-31.
10. Bravata DM, Ho SY, Meehan TP, et al. Readmission and death after hospitalization for acute ischemic stroke: 5-year follow-up in the medicare population. *Stroke.* (2007) 38(6):1899-904.
11. Kuan-Ying Wu S-CW, Yen-Ni Hung, Chun-Chen Wu,, Li-Chan Lin H-HH. The need for post-acute care for stroke patients in Taiwan. *Taiwan Journal of Public Health.* (2012) 31(3):251-62.
12. Chen LK, Chen YM, Hwang SJ, et al. Effectiveness of community hospital-based post-acute care on functional recovery and 12-month mortality in older patients: a prospective cohort study. *Ann Med.* (2010) 42(8):630-6.
13. Lee WJ, Peng LN, Cheng YY, et al. Effectiveness of short-term interdisciplinary intervention on postacute patients in Taiwan. *J Am Med Dir Assoc.* (2011)

12(1):29-32.
14. Lee WJ, Chou MY, Peng LN, et al. Predicting clinical instability of older patients in post-acute care units: a nationwide cohort study. *Geriatr Gerontol Int.* (2014) 14(2):267-72.
15. Wang CY, Chen YR, Hong JP, et al. Rehabilitative post-acute care for stroke patients delivered by per-diem payment system in different hospitalization paths: A Taiwan pilot study. *Int J Qual Health Care.* (2017) 29(6):779-84.
