# Supplementary material for: Cost Utility Analysis of Multidisciplinary Postacute Care for Stroke: A Prospective Six-Hospital Cohort Study
Source: Front Cardiovasc Med. 2022 Mar 30;9:826898. doi: 10.3389/fcvm.2022.826898 (PMC9007246; doi:10.3389/fcvm.2022.826898)

Supplementary eFigure 1

(120:120)

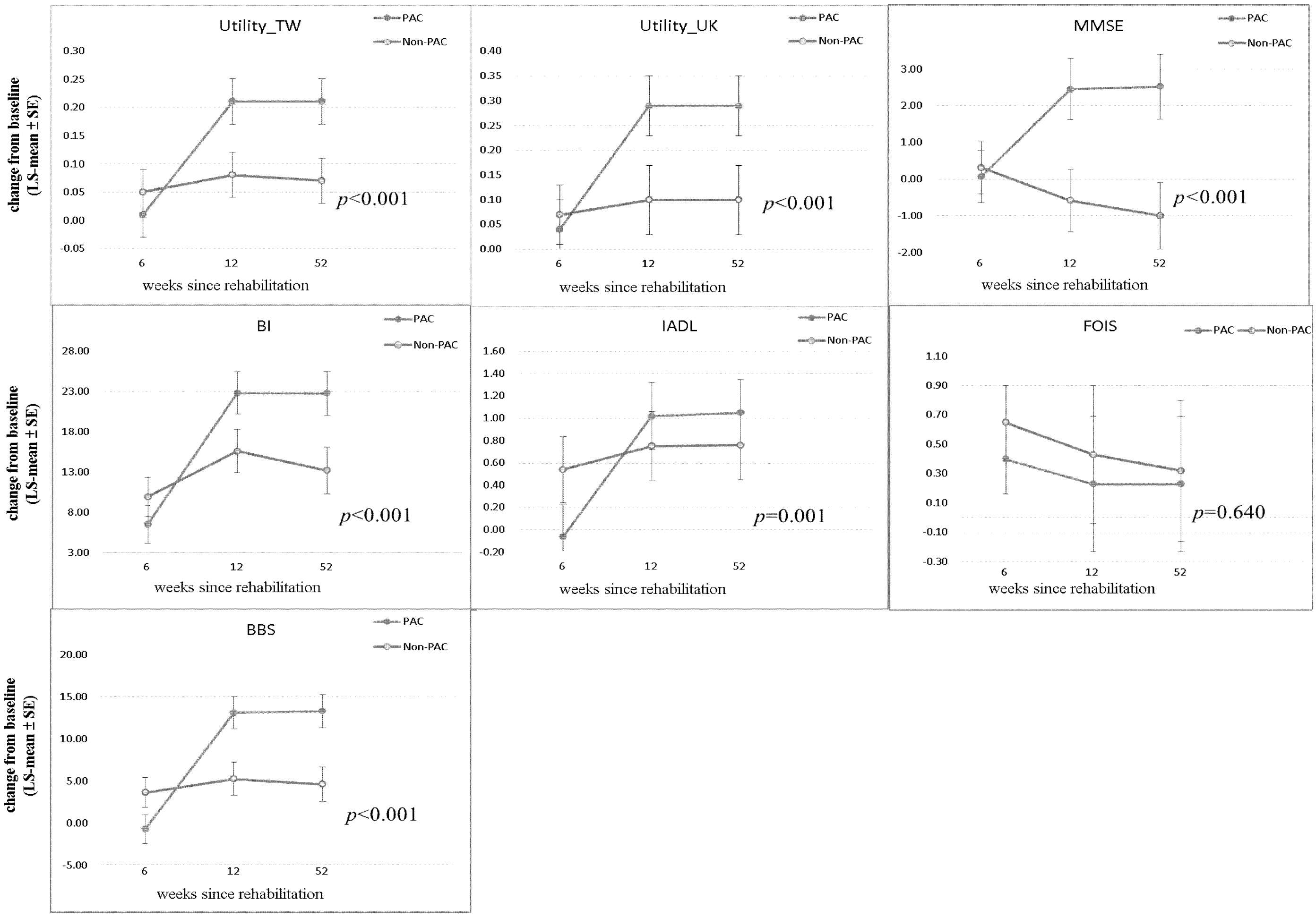

Supplementary eFigure 2
(62:62)

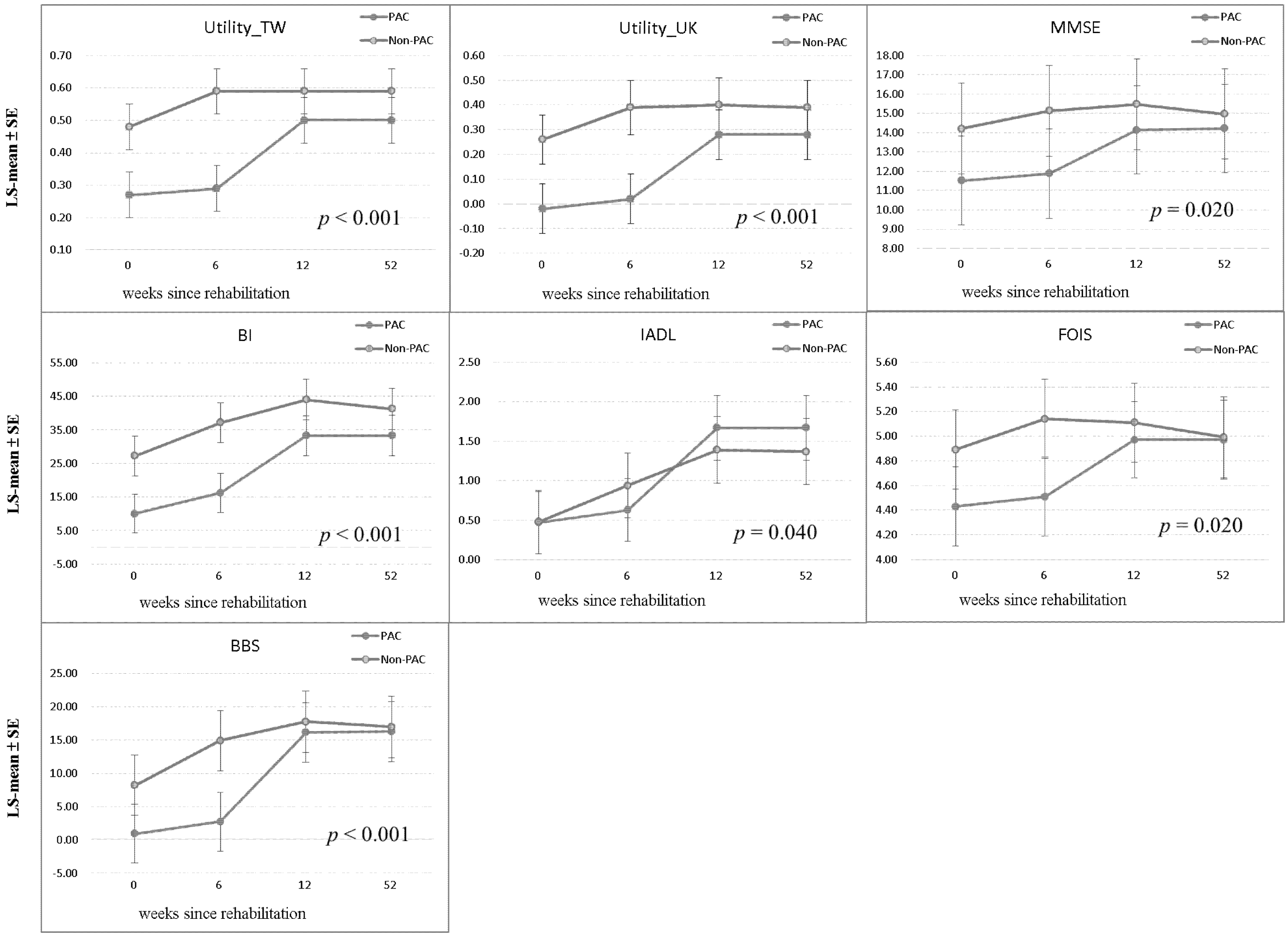

Supplementary eFigure 3

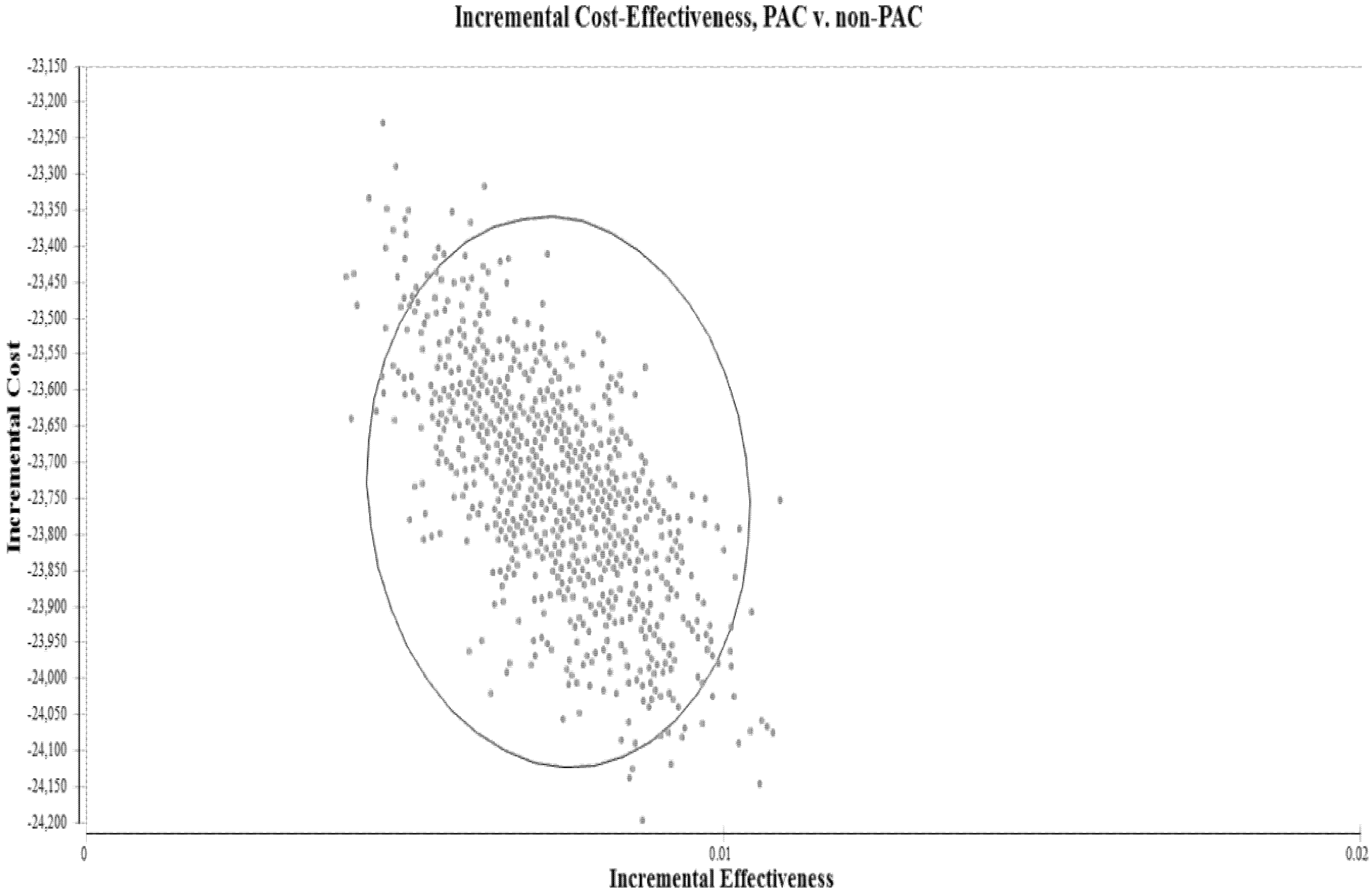

Supplementary eFigure 4

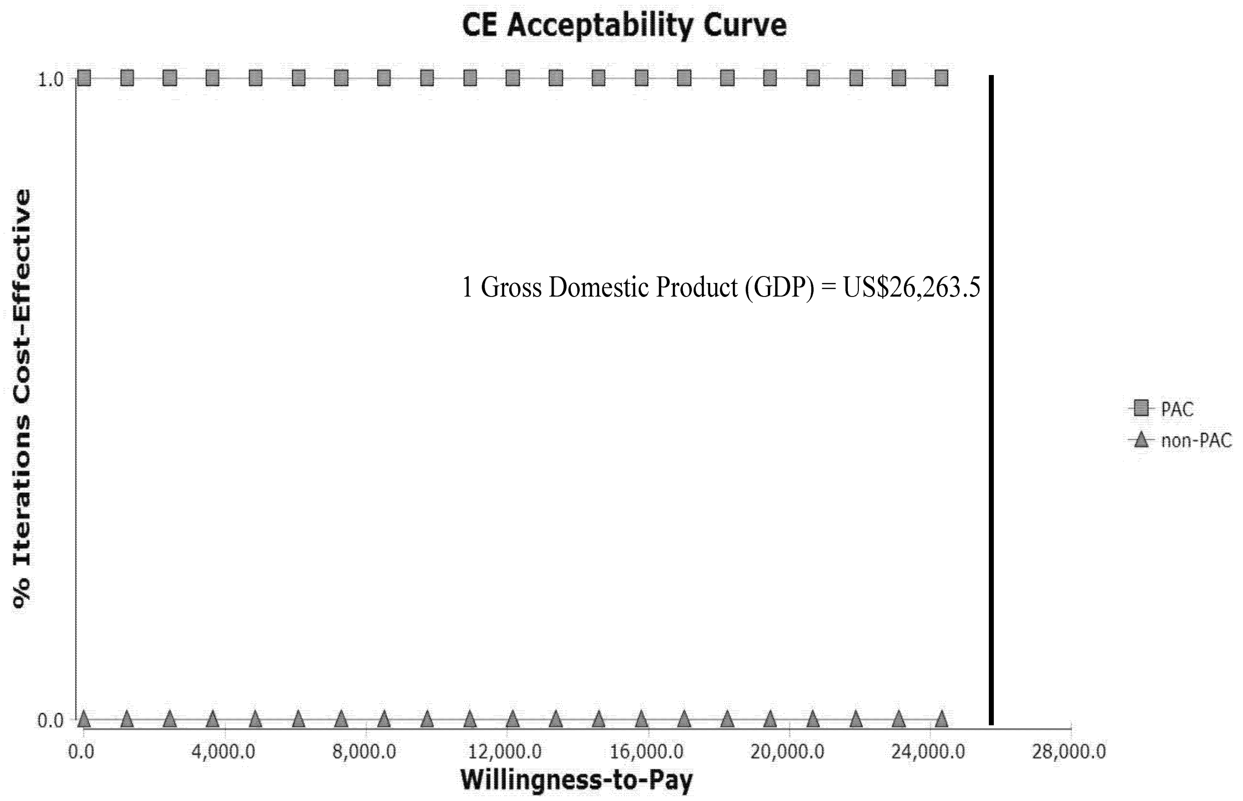

Supplementary eFigure 5
(164:82)

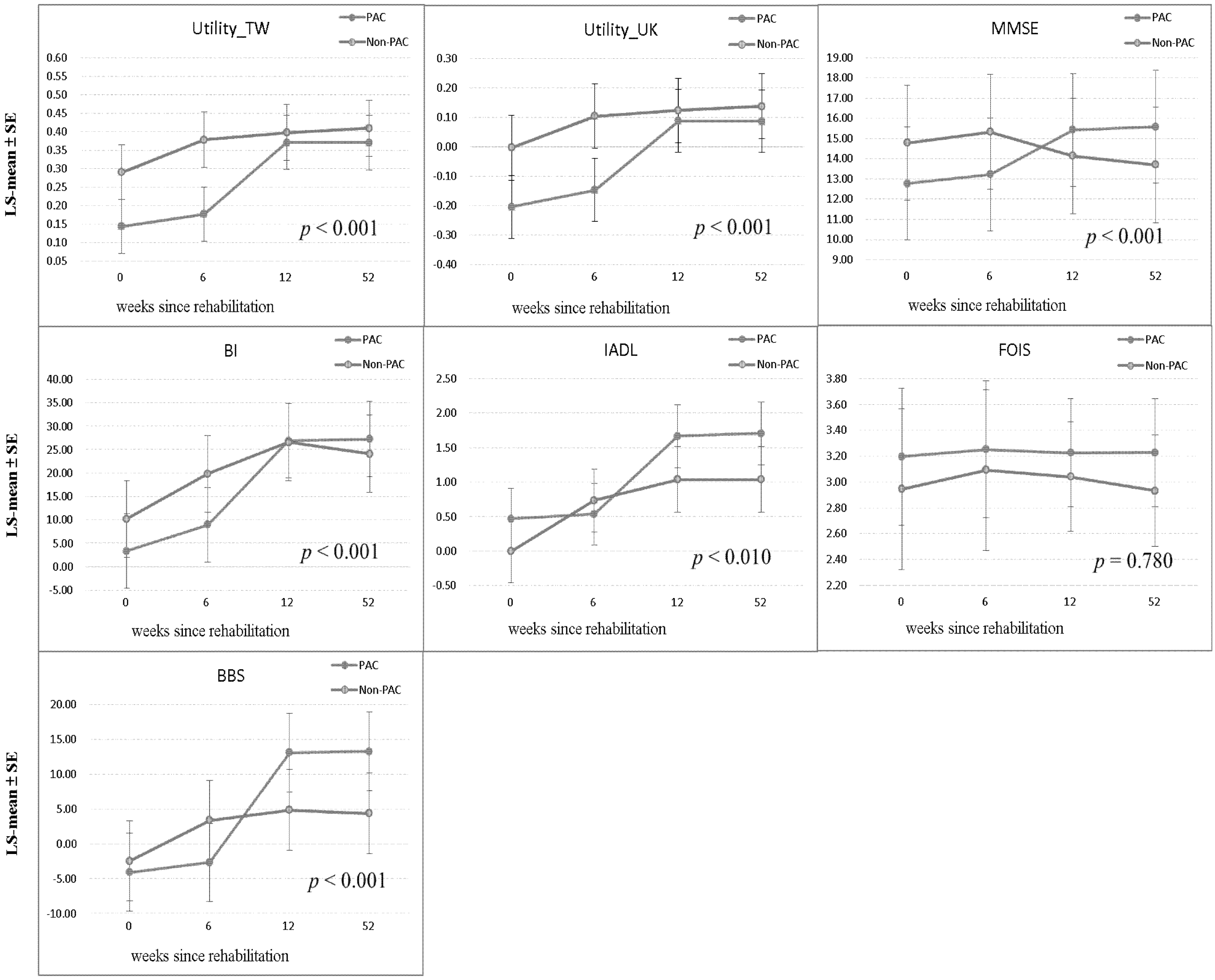

Supplement: Supplementary Figure 1 — Comparison by LS-mean ± SE of each functional status measure between PAC and non-PAC groups, after controlling for baseline, week 6 and 12, and 1 year data and comparison of trend in each functional status measure between the groups. Utility_TW, Utility (Taiwan); Utility_UK, Utility (United Kingdom); MMSE, Mini-Mental State Examination; BI, Barthel index; IADL, Instrumental Activities of Daily Living; FOIS, Functional Oral Intake Scale; BBS, Berg Balance Scale; PAC, postacute care; LS-mean, least squares mean; SE, standard error. P values for trend in each functional status measure between the groups (120:120). [file Data_Sheet_2.pdf]
